# Urinary Polycyclic Aromatic Hydrocarbons and Advanced Cardiovascular‐Kidney‐Metabolic Syndrome: Inflammatory‐Nutritional Pathways as Mediators

**DOI:** 10.1155/mi/5585916

**Published:** 2026-06-02

**Authors:** Yifeng Huang, Youxia Zhao, Qizhang Man, Song Liu, Ying Yang, Jinfeng Wen, Lei Fan, Hao Xie, Keyun Zhang, Jing Wang

**Affiliations:** ^1^ Nephrology Department of Ganzhou People’s Hospital, Ganzhou, Jiangxi, China, gzsrmyy.com; ^2^ School of Stomatology, Southern Medical University, Guangzhou, 510515, Guangdong, China, fimmu.com; ^3^ Department of Medical Imaging Center, Nan Fang Hospital, Southern Medical University, No. 1838 Guangzhou Avenue North, Guangzhou, 510515, Guangdong, China, fimmu.com; ^4^ The First School of Clinical Medicine, Southern Medical University, Guangzhou, 510515, Guangdong, China, fimmu.com; ^5^ School of Laboratory Medicine and Biotechnology, Southern Medical University, Guangzhou, 510515, Guangdong, China, fimmu.com; ^6^ Division of Orthopaedic Surgery, Department of Orthopaedics, Nanfang Hospital, Southern Medical University, Guangzhou, 510515, Guangdong, China, fimmu.com; ^7^ Department of Joint Sport Medicine, The Fist Affiliated Hospital of Hunan Medical College, 225 Yushi Road, Huaihua, 418000, Hunan, China

**Keywords:** CKM syndrome, environmental exposure, mediation analysis, polycyclic aromatic hydrocarbons, red blood cell distribution width-to-albumin ratio

## Abstract

**Background:**

Polycyclic aromatic hydrocarbons (PAHs) are widespread environmental pollutants linked to adverse health outcomes. Their role in the different statuses of Cardiovascular‐Kidney‐Metabolic (CKM) syndrome remains poorly understood.

**Methods:**

A cross‐sectional analysis of 11,043 participants was conducted using data from NHANES. Urinary PAH levels were measured, and advanced CKM status was assessed using established diagnostic criteria. Mediation analysis was performed to evaluate the role of the red blood cell distribution width‐to‐albumin ratio (RAR).

**Results:**

Higher urinary levels of PAHs, particularly 2‐NAP and 2‐FLU, were significantly associated with advanced CKM syndrome (*p* < 0.05). Exposure‐response relationships and threshold effects were observed for these key PAHs. Mediation analysis identified RAR as a novel biomarker, explaining 6.13%–37.81% of the association depending on the specific metabolite.

**Conclusions:**

This study reveals that PAHs are independently and jointly associated with advanced CKM status, with RAR serving as a critical mediator. These findings underscore the importance of reducing environmental PAH exposure and highlight RAR as a potential biomarker for early detection and risk stratification.

## 1. Introduction

The Cardiovascular‐Kidney‐Metabolic (CKM) syndrome, as defined by the American Heart Association (AHA) in 2023, represents a systemic, progressive condition arising from the intricate interplay among metabolic disorders, chronic kidney disease (CKD), and cardiovascular disease (CVD) [1, 2]. This syndrome significantly exacerbates disease burden and mortality through complex pathophysiological mechanisms, including hyperglycemia and impaired renal and cardiac function [3]. CKM syndrome affects a substantial proportion of the population, with studies revealing that over 25% of U.S. adults were diagnosed with CKM between 2015 and 2020 [4], and ~90.8% met the criteria for stages 0–3 [5]. Furthermore, this syndrome is associated with significant healthcare costs, accounting for over 75% of total expenditures for related conditions [4, 6]. The AHA has emphasized the importance of early identification and intervention, particularly in stages 0–3, to prevent cardiovascular events and improve outcomes, as these preclinical stages are critical in halting the progression of CKM and reducing morbidity and mortality [7, 8].

An increasing body of epidemiological evidence indicates that environmental pollution is associated with multisystem diseases throughout the body [9–11]. Polycyclic aromatic hydrocarbons (PAHs) are chemical compounds with more than two fused aromatic rings, mainly produced through the incomplete combustion of materials such as coal, petroleum, rubber, plastics, wood, and tobacco [12]. Due to their lipophilic properties, PAHs are easily absorbed into the human body via inhalation, ingestion, and skin contact [13, 14]. Their metabolites, excreted through urine and feces, serve as biomarkers for exposure [15]. Chronic PAH exposure is associated with oxidative stress, metabolic syndrome, CVDs, and cancers [16, 17]. Mechanistically, PAHs induce oxidative stress, endothelial dysfunction, and inflammation, contributing to disease progression [18, 19]. Additionally, hydroxylated PAHs disrupt hormonal pathways, exhibiting estrogenic, antiestrogenic [20], antiandrogenic [21], and thyroid receptor antagonistic effects [22]. These endocrine‐disrupting properties, along with the bioaccumulation of PAHs in adipose tissue, liver, and kidneys [23], highlight their role in metabolic dysregulation and organ toxicity, making them critical environmental risk factors for complex conditions such as CKM syndrome.

The red blood cell distribution width‐to‐albumin ratio (RAR) is a novel biomarker that integrates markers of systemic inflammation and nutritional status [24]. RDW reflects oxidative stress and inflammation [25], while albumin indicates nutritional and inflammatory states [26]. Together, RAR offers a comprehensive measure of inflammatory stress and metabolic dysfunction, with studies linking it to outcomes in CVDs, CKD, and stroke [27, 28]. The significance of RAR is particularly apparent in the context of PAHs, which are known to induce oxidative stress, inflammation, and metabolic dysregulation. These pathological processes intersect with the mechanisms encompassed by RAR, indicating its potential role as a mediator in the health effects associated with PAH exposure.

The purpose of our research is to use NHANES data from 2001 to 2016 to explore the association between PAHs and advanced CKM syndrome in the population and to investigate the mediating role of RAR in this process, providing valuable insights into how environmental exposure such as PAHs leads to inflammation‐driven metabolic disorders.

## 2. Methods

### 2.1. Study Population

The NHANES is conducted by the Centers for Disease Control and Prevention’s National Center for Health Statistics to assess the health and nutritional status of the civilian, noninstitutionalized U.S. population. The survey operates under the ethical oversight of the NCHS Research Ethics Review Board and obtains written informed consent from all participants [8, 29]. The complete survey data, documentation, and analytical guidelines are publicly accessible through the NHANES website (https://wwwn.cdc.gov/nchs/nhanes/Default.aspx).

**Figure 1:**
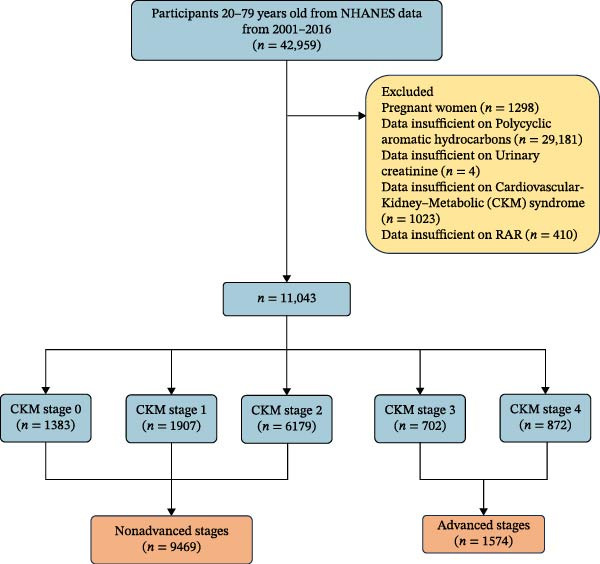
Flow diagram of the screening and enrollment of study participants.

We combined data from eight survey cycles spanning 2001–2016. The analysis incorporated six PAHs and included a total sample population of 11,043 adults aged 20 years and older. During participant selection, pregnant women at the time of examination (*n* = 1298), those without PAH measurement data (*n* = 29,181), those without urinary creatinine data (*n* = 4), those with missing CKM status (*n* = 1023), and those with insufficient RAR data (*n* = 410) were excluded, as illustrated in Figure 1.

### 2.2. Main Exposure Measurement: Urinary PAH Metabolites

Urinary PAH metabolites were analyzed in spot urine samples collected during the NHANES physical examination, following rigorous collection and processing protocols. Urine specimens were collected in sterile polypropylene tubes, enzymatically hydrolyzed, and stored at −20°C prior to analysis. PAH metabolites were determined by capillary gas chromatography joint with high‐resolution mass spectrometry (GC/HRMS) (2001–2008 wave), isotope dilution capillary gas chromatography‐tandem mass spectrometry (GC–MS/MS) (2009–2012), and isotope dilution high performance liquid chromatography‐tandem mass spectrometry (online SPE‐HPLC‐MS/MS) (2013–2016) [30]. All methods underwent rigorous quality control and calibration to ensure comparability across survey cycles, as detailed in the NHANES laboratory procedures. The six metabolites included in this study are 1‐hydroxynaphthalene (1‐NAP), 2‐hydroxynaphthalene (2‐NAP), 2‐hydroxyfluorene (2‐FLU), 3‐hydroxyfluorene (3‐FLU), 1‐hydroxyphenanthrene (1‐PHE), and 1‐hydroxypyrene (1‐PYR). Following NHANES/CDC standard laboratory procedures, concentrations below the limit of detection (LOD) were assigned a value equal to the LOD divided by the square root of two (LOD/√2). The primary data processing was performed by NHANES. Detailed detection frequencies and LOD values for each PAH metabolite are summarized in Table S18. Further details on analytical methods and quality control protocols are available on the NHANES website (https://wwwn.cdc.gov/nchs/nhanes/Default.aspx). Each urine PAH metabolite was corrected with creatinine (Cr) to take into account urine dilution [31]. Each Cr‐corrected urine PAH metabolite underwent natural logarithmic transformation to mitigate the influence of extreme values and improve the conformity to the normal distribution during regression and mediation analysis. The Cr correction formula used is as follows:

Corrected PAH ng/g creatinine=measured PAH ng/Lurinary creatinine mg/dL×0.01.

### 2.3. Definition of CKM Syndrome Stages 0–4

In accordance with the 2023 AHA Presidential Advisory, CKM syndrome is defined as a systemic disorder characterized by the pathophysiological interplay among metabolic risk factors, CKD, and the cardiovascular system. These clinical components were assessed using established guidelines. CKD is determined using the CKD‐EPI creatinine equation [32], with an estimated glomerular filtration rate (eGFR) of less than 60 mL/min/1.73 m^2^, or a urinary albumin‐to‐creatinine ratio (UACR) exceeding 30 mg/g, serving as diagnostic thresholds. CVDs are judged by past medical history. The PREVENT formula is used to estimate the 10‐year risk of CVDs in subclinical CVDs (Table S2). Metabolic syndrome (MS), on the other hand, is identified based on the National Cholesterol Education Program Adult Treatment Panel III (NCEP‐ATP III) criteria [33], which require the presence of at least three of the following risk factors: central obesity, as defined by a waist circumference ≥102 cm for men or ≥88 cm for women; elevated triglycerides (≥150 mg/dL); low HDL cholesterol (<40 mg/dL for men or <50 mg/dL for women); hypertension (systolic blood pressure ≥130 mmHg or diastolic blood pressure ≥80 mmHg); and hyperglycemia (fasting plasma glucose ≥100 mg/dL or the use of antidiabetic medications) (Table S1).

In the 2023 AHA CKM staging framework, participants are categorized into stages CKM 0–4 (Table S3). To specifically evaluate the impact of PAHs on the progression from risk accumulation to clinical disease, the study population was dichotomized into two groups: the “Nonadvanced CKM” group (Stages 0–2), representing healthy individuals (Stage 0) or the prestage of accumulation of metabolic risk factors; and the “Advanced CKM” group (Stages 3–4), representing the phase of subclinical or established CVD and significant target organ damage. This classification method has been applied in previous studies, as these advanced stages effectively identify individuals who have established CVD or are at a high risk for CVD [5, 34].

### 2.4. Assessment of Covariates

This study considered a variety of covariates, including age, sex, race, education, marital status, poverty income ratio (PIR), body mass index (BMI), smoking and alcohol use, physical activity levels, various diseases and cardiovascular health conditions, as well as some biochemical test indicators.

Participants’ smoking status was categorized through questionnaire responses: those who report their current use of tobacco products and those who have smoked more than 100 cigarets in their lifetime are designated as smokers. The study defined “alcohol use” based on whether participants reported consuming a minimum of 12 alcoholic beverages annually or had exceeded 12 drinks throughout their lives. This study evaluated physical activity through questionnaire‐based weekly physical activity participation data. The physical activity measurements were converted into metabolic equivalent (MET) minutes of moderate to vigorous physical activity per week [35]. We conducted a directed acyclic graph analysis on the covariates (Figure S1) and ensured that the variance inflation factor of each covariate was less than 5 (Figure S2).

### 2.5. Statistical Analysis

In accordance with NHANES analytic guidelines, analyses accounted for the survey design by incorporating the appropriate urinary PAH laboratory subsample weights, strata (SDMVSTRA), and PSUs (SDMVPSU). For pooled analyses across the 2001–2016 cycles, we constructed multicycle sampling weights following NHANES recommendations for combining continuous NHANES cycles by rescaling the 2‐year subsample weight by the number of survey cycles included. Firstly, in the baseline information presentation, continuous variables are expressed as weighted Median and 25% and 75% quantiles, while categorical variables are expressed as counts and corresponding percentages. The intergroup comparisons were conducted using a design‐based Mann–Whitney *U* test and a chi‐square test for the analysis of continuous variables and categorical variables. Second, we used binary classification weighted logistic regression to evaluate the association between PAHs and CKM status, as well as established three models. The first crude model did not adjust for confounding factors, and the second model adjusted for demographic factors. The third model further adjusted for BMI, smoking and alcohol use, aspartate aminotransferase (AST), glutamate aminotransferase (ALT), and physical activity level (MET). Thirdly, restricted cubic spline (RCS) analysis was adopted to visualize and explore the potential nonlinear associations between various PAHs and CKM status. In order to determine the best number of nodes, Akaike information criteria (AIC) and Bayesian information criteria (BIC) for different number of nodes (3–6) were calculated for model selection. Given the large size of our cohort, in order to achieve a better balance between model complexity and generalization ability, we preferentially refer to BIC for junction selection to reduce the risk of overfitting and improve the interpretability of the model. The calculation results of AIC and BIC as well as the final number of settlements are detailed in the Table S6. Subsequently, we developed piecewise logistic regression models incorporating the identified threshold values. To evaluate model fit, we conducted log‐likelihood ratio testing between conventional linear and piecewise regression approaches.

Fourth, we performed BKMR‐P and Qgcomp analyses to investigate the relationship between urinary PAH mixtures and CKM status. Due to the current software limitations of the R packages used for advanced modeling (bkmrhat, qgcomp, and mediation), fully incorporating the complex multistage survey design (sample weight, PSUs, and strata) into these specific algorithms is methodologically not supported in current software implementations. Therefore, consistent with common practice in epidemiologic applications when full survey integration is not available, the mixture models and the mediation analyses were performed without the full survey design parameters. To justify the use of these methods, we first calculated the Spearman rank correlations among the various PAH metabolites. BKMR‐P is designed to handle multicollinearity in datasets with many variables and high intercorrelation, making it well‐suited for estimating the impact of combined PAH exposures. Qgcomp provides an enhanced estimation framework and accommodates nonlinear associations within mixtures. BKMR‐P produces nonlinear plots by comparing predicted values when all exposure variables are set to a given percentile with those when they are fixed at the 50th percentile, offering an effective means to visualize the joint effects of PAH mixtures on CKM status. In contrast, Qgcomp yields more precise quantitative estimates. Moreover, BKMR‐P reports the posterior inclusion probability for each PAH metabolite, whereas Qgcomp calculates the weight contribution of each mixture component. Using both BKMR‐P and Qgcomp strengthens the robustness and interpretability of our analysis.

Fifth, a statistical mediation analysis was conducted to explore whether RAR potentially mediated the association between PAHs and the risk of advanced CKM status. According to the mediator’s criteria [36], in addition to the positive correlation between X (logarithmic transformed PAHs) and Y (risk of the advanced CKM status), it is also necessary to observe statistically significant associations between X and M (RAR) as well as between M and Y. Therefore, we also conducted a generalized linear model (GLM) to examine the associations between PAHs and RAR, as well as between RAR and the advanced CKM status risks. Due to the narrow range of numerical variation of RAR, in order to avoid an inflated OR, we standardized RAR in the subsequent regression analysis. If these criteria are met, a mediating effect model will be conducted to assess the mediating ratio (%) of RAR in the relationship between PAHs and the risk of the advanced CKM status.

Sixth, subgroup analysis was conducted to assess age (<65 or ≥65), sex (male or female), race (Mexican American, non‐Hispanic Black, non‐Hispanic White, other Hispanics, or other races), education (above high school, high school or equivalent, or less than high school), smoking (yes or no), alcohol use (yes or no), and physical activity level (sufficient activity or insufficient activity).

Finally, to evaluate the robustness of the association between PAHs and the advanced CKM status, we conducted several sensitivity analyses to validate our main findings. (1) We have adjusted the interview years of the respondents and RAR. (2) Analyze the missing covariates after multiple imputation using the “mice” package. (3) We used non‐Cr‐corrected PAHs as exposure and creatinine as a covariate in the model.

All analyses were conducted using Version R 4.4.1, and a two‐sided *p*‐value less than 0.05 was considered significant.

## 3. Result

### 3.1. Baseline Characteristics of Study Samples

The study included 11,043 participants (median age 44 years, 50% male) stratified into nonadvanced (*n* = 9469, 89%) and advanced stages (*n* = 1574, 11%). Advanced stage participants were older (median 65 years), predominantly male (61%), and exhibited lower socioeconomic status with reduced education levels and PIRs. This group demonstrated higher BMI (median 30) and significantly increased prevalence of cancer (17%), diabetes (41%), hypertension (81%), CVD (60%), and smoking (64%), alongside reduced physical activity levels. Laboratory analysis revealed lower albumin (42 g/L) and elevated AST and RAR in advanced stages, with comparable ALT levels between groups. All differences were statistically significant except alcohol consumption and ALT levels (Table 1).

**Table 1:** Baseline characteristics across CKM status of participants in NHANES 2001–2016.

Characteristic	Overall	Nonadvanced stages	Advanced stages	*p*‐Value ^∗^
	*N* = 11,043 (100%)^a^	*N* = 9469 (89%)^a^	*N* = 1574 (11%)^a^	
Sex, *n* (%)				**<0.001**
Female	5446 (50%)	4831 (51%)	615 (39%)	
Male	5597 (50%)	4638 (49%)	959 (61%)	
Age, years	44 (32, 56)	42 (30, 53)	65 (56,71)	**<0.001**
Race, *n* (%)				**<0.001**
Mexican American	1998 (8.5%)	1760 (8.9%)	238 (5.7%)	
Non‐Hispanic black	2308 (11%)	1924 (11%)	384 (12%)	
Non‐Hispanic white	4755 (68%)	4020 (68%)	735 (73%)	
Other Hispanic	979 (5.6%)	850 (5.8%)	129 (3.9%)	
Other race	1003 (6.7%)	915 (6.9%)	88 (4.7%)	
Education, *n* (%)				**<0.001**
Greater than high school	5590 (61%)	4935 (62%)	655 (50%)	
High school or equivalent	2468 (23%)	2083 (23%)	385 (26%)	
Less than high school	2658 (16%)	2126 (15%)	532 (24%)	
Marital status, *n* (%)				0.4
Having a partner	6640 (65%)	5691 (65%)	949 (66%)	
Without a partner	4233 (35%)	3610 (35%)	623 (34%)	
PIR	3.12 (1.50, 5.00)	3.20 (1.54, 5.00)	2.50 (1.27, 4.45)	**<0.001**
BMI (kg m^2^)	28 (24, 32)	27 (24, 32)	30 (26, 34)	**<0.001**
Cancer patients, *n* (%)				**<0.001**
Yes	749 (7.6%)	499 (6.4%)	250 (17%)	
No	9960 (92%)	8640 (94%)	1320 (83%)	
CVD, *n* (%)				**<0.001**
Yes	872 (6.6%)	0 (0%)	872 (60%)	
No	9806 (93%)	9115 (100%)	691 (40%)	
Diabetes, *n* (%)				**<0.001**
Yes	1631 (11%)	904 (7.8%)	727 (41%)	
No	9412 (89%)	8565 (92%)	847 (59%)	
Hypertension, *n* (%)				**<0.001**
Yes	4126 (34%)	2791 (28%)	1335 (81%)	
No	6917 (66%)	6678 (72%)	239 (19%)	
Smoking, *n* (%)				**<0.001**
Yes	4933 (46%)	3960 (44%)	973 (64%)	
No	5838 (54%)	5237 (56%)	601 (36%)	
Alcohol use, *n* (%)				0.9
Yes	7651 (90%)	6474 (90%)	1177 (90%)	
No	1165 (10%)	995 (10%)	170 (10%)	
Albumin (g/L)	43 (41,45)	43 (41, 45)	42 (40, 44)	**<0.001**
Red blood cell distribution width	12.70 (12.20, 13.40)	12.70 (12.20, 13.30)	13.10 (12.50, 13.80)	**<0.001**
AST (U/L)	23 (20,28)	23 (20, 28)	24 (20, 29)	**0.003**
ALT (U/L)	22 (17, 30)	22 (17, 30)	22 (18, 29)	0.10
MET	1879 (720, 4560)	1920 (750, 4,671)	1440 (600, 3, 443)	**<0.001**

*Note:* Bolded *p*‐values indicate statistical significance.

Abbreviations: ALT, glutamate aminotransferase; AST, aspartate aminotransferase; BMI, body mass index; CVD, cardiovascular disease; PIR, the ratio of family income to poverty.

^a^ *n* (unweighted) (%); Median (Q1, Q3).

^∗^Pearson’s *X* ^2^: Rao and Scott adjustment; Design‐based Mann–Whitney *U* test.

The distribution of six types of PAHs and RAR is shown in Table 2. The average concentrations of 1‐NAP, 2‐NAP, 2‐FLU, 3‐FLU, 1‐PHE, and 1‐PYR are 7.76 ± 1.45, 8.38 ± 1.01, 5.66 ± 1.05, 4.77 ± 1.23, 4.87 ± 0.75, and 4.66 ± 0.97, respectively, and the average value of RAR is 0.31 ± 0.04. Except for 1‐PYR and 2‐NAP, the other PAH metabolites all began to show a downward trend in the 2005–2006 cycle, as shown in Table S5. The sample density curve indicates that 1‐NAP and 2‐FLU were higher in the advanced stages group than in the nonadvanced stages group (*p*‐value < 0.05) (Figure S3).

**Table 2:** Descriptive statistics of PAHs and RAR.

Variable	Mean	SD	P10	P25	P50	P75	P90
1‐Hydroxynapthalene	7.76	1.45	6.15	6.69	7.48	8.73	9.68
2‐Hydroxynapthalene	8.38	1.01	7.08	7.62	8.35	9.14	9.74
2‐Hydroxyfluorene	5.66	1.04	4.54	4.88	5.38	6.32	7.28
3‐Hydroxyfluorene	4.77	1.23	3.45	3.86	4.42	5.57	6.75
1‐Hydroxyphenanthrene	4.87	0.75	3.99	4.38	4.83	5.33	5.81
1‐Hydroxypyrene	4.65	0.97	3.47	4.03	4.63	5.26	5.88
RAR	0.31	0.04	0.26	0.28	0.30	0.33	0.36

Abbreviations: RAR, red blood cell distribution width albumin ratio; SD, standard deviation.

### 3.2. Independent Associations Between Urinary PAH Metabolites and CKM Status

**Figure 2:**
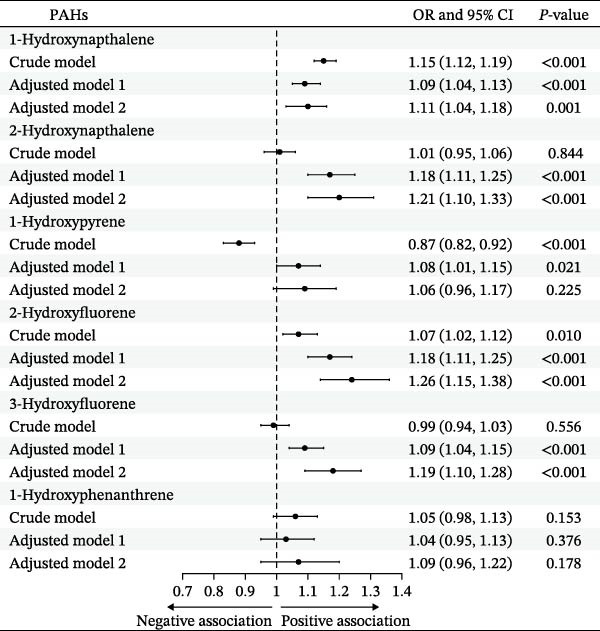
The association between six PAHs and the advanced CKM status. The crude model did not adjust for confounding factors, and Model 1 adjusted for demographic factors. The Model 2 further adjusted for BMI, smoking and alcohol use, aspartate aminotransferase (AST), glutamate aminotransferase (ALT), and physical activity level (MET).

Figure 2 shows the odds ratio (OR) and 95% confidence intervals (95% CI) of various PAHs (after Cr correction and Ln conversion). We observed that higher urinary levels of 1‐NAP, 2‐NAP, 2‐FLU, and 3‐FLU were significantly associated with advanced CKM status, and these associations remained robust after adjusting for potential confounders. The OR value and 95%CI of PAHs in the fully adjusted model were 1‐NAP (OR = 1.11, 95% CI: 1.04–1.18), 2‐NAP (OR = 1.21, 95% CI: 1.10–1.33), 2‐FLU (OR = 1.26, 95% CI: 1.15–1.38), and 3‐FLU (OR = 1.19, 95% CI: 1.10–1.28).

To further explore potential nonlinear relationships, we conducted RCS analyses. By using RCS with three nodes (Figure 3), we visualized the exposure‐response relationship between each PAH metabolite and CKM status and found that all PAH metabolites were nonlinearly correlated with the advanced CKM status, except for 1‐NAP and 2‐NAP (*P*‐nonlinear = 0.259 and *P*‐nonlinear = 0.195). A recursive segmentation algorithm, combined with two‐stage logistic regression, was applied to the six PAH metabolites and calculated that the segmentation points of 2‐FLU, 3‐FLU, 1‐PHE, and 1‐PYR were 6.35, 4.17, 4.59, and 5.09, respectively. After the segmentation points, PAH metabolites showed a significant positive correlation with advanced CKM. However, there was no obvious threshold effect between the metabolites 1‐NAP and 2‐NAP (all *P* for the likelihood test >0.05). This is consistent with the results of RCS (Table 3).

Figure 3The exposing‐response relationships between six types of PAHs and advanced CKM status. (A) 1‐Hydroxynapthol; (B) 2‐Hydroxynapthol; (C) 1‐Hydroxypyrene; (D) 2‐Hydroxyfluorene; (E) 3‐Hydroxyfluorene; (F) 1‐Hydroxyphenanthrene. The model adjustment is consistent with that of Model 2.

**Figure:**
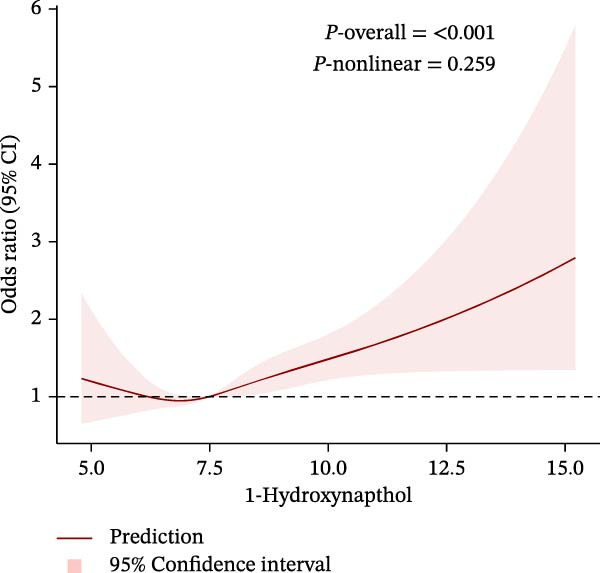
(A)

**Figure:**
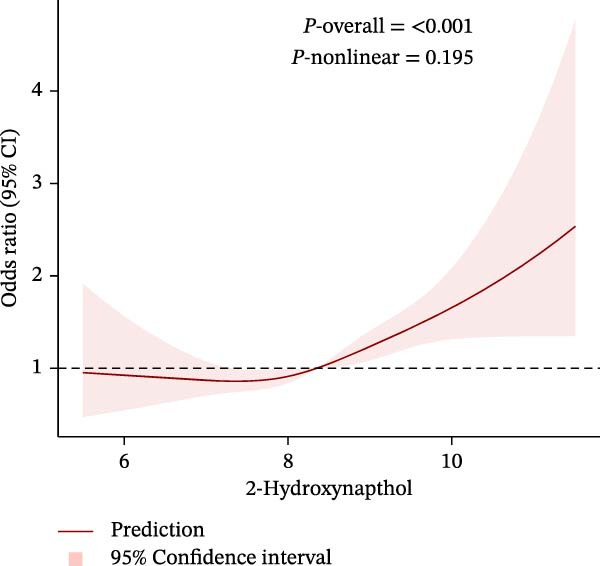
(B)

**Figure:**
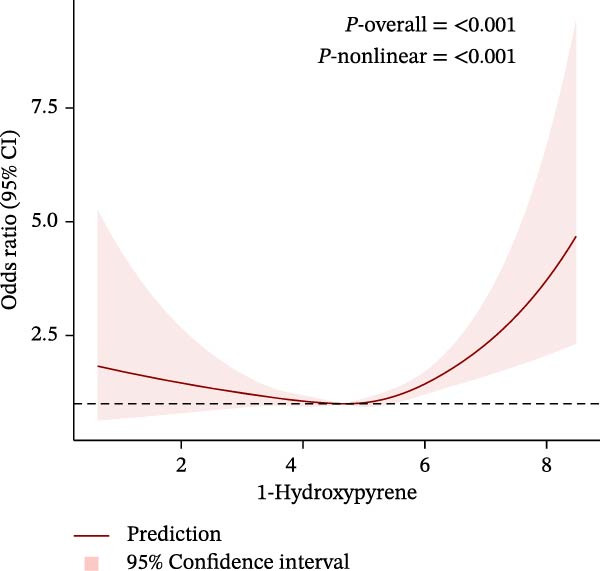
(C)

**Figure:**
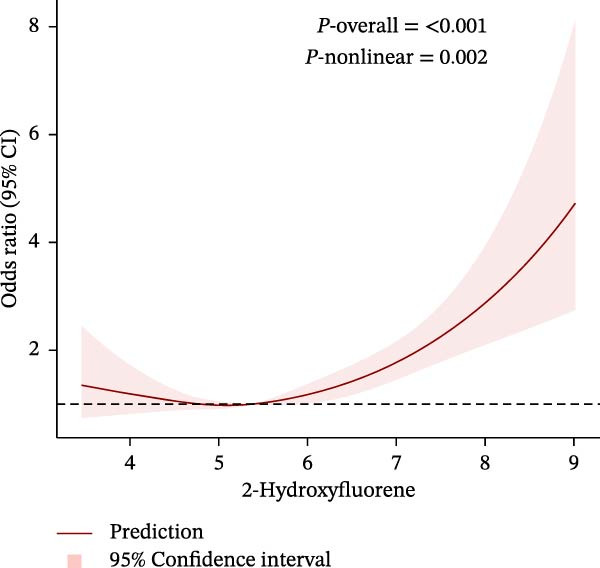
(D)

**Figure:**
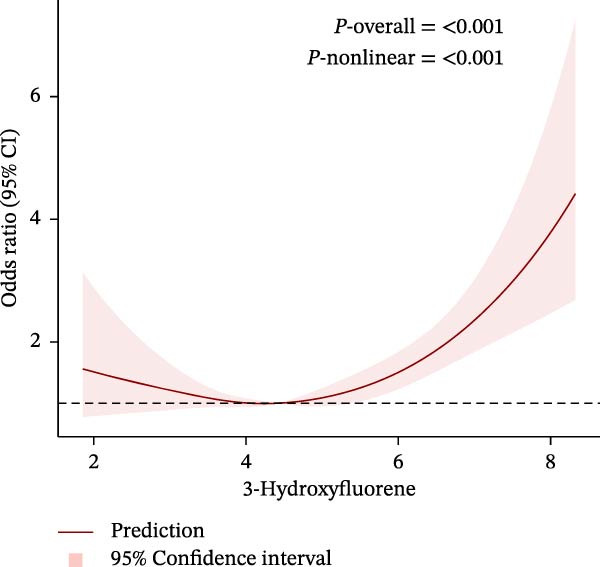
(E)

**Figure:**
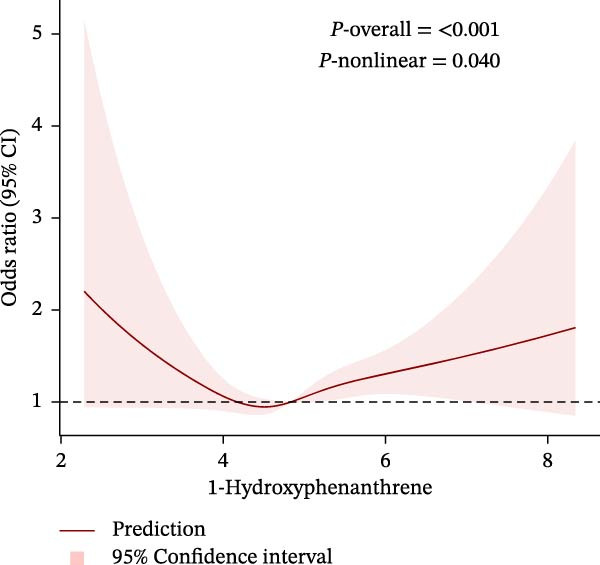
(F)

**Table 3:** Odds ratios of PAHs with CKM status by weighted 2‐piecewise multivariate logistic regression.

Variable	Cut points	<Cut points^a^	≥Cut points^a^	*p* for likelihood test
1‐Hydroxynapthalene	9.90	1.16 (1.07, 1.26) <0.001	0.94 (0.76, 1.13) 0.510	0.060
2‐Hydroxynapthalene	8.48	1.05 (0.88, 1.26) 0.575	1.40 (1.16, 1.67) <0.001	0.073
2‐Hydroxyfluorene	6.35	1.06 (0.91, 1.23) 0.485	1.66 (1.33, 2.08) <0.001	**0.007**
3‐Hydroxyfluorene	4.17	0.78 (0.60, 1.01) 0.061	1.33 (1.20, 1.48) <0.001	**0.001**
1‐Hydroxyphenanthrene	4.59	0.74 (0.53, 1.03) 0.070	1.24 (1.05, 1.46) 0.008	**0.016**
1‐Hydroxypyrene	5.09	0.85 (0.73, 0.98) 0.030	1.50 (1.23, 1.83) <0.001	**<0.001**

*Note:* Model was adjusted for age, sex, race, education, marital status, poverty income ratio (PIR), body mass index (BMI), smoking and alcohol use, aspartate aminotransferase (AST), glutamate aminotransferase (ALT), and physical activity level (MET). Bolded *p*‐values indicate statistical significance.

^a^Odds ratio (95% CI) *p*‐value.

### 3.3. Joint Associations Between Urinary PAH Metabolites and CKM Status

We employed Spearman correlation analyses. The correlation coefficients indicated that the concentrations of the six urinary PAH metabolites were significantly correlated (Spearman: 0.34–0.92), as shown in Figure S4.

**Figure 4:**
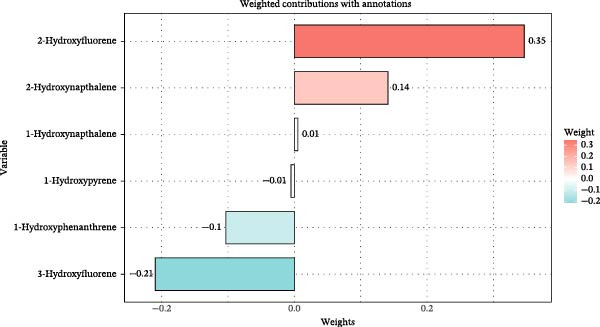
The combined exposure to a mixture of polycyclic aromatic hydrocarbons and the advanced CKM status (weight) was evaluated by quantile g calculation. The model adjustment is consistent with that of Model 2.

Given this high correlation, we employed the Qgcomp and BKMR‐P models to explore the impact of the combined effect of multiple PAHs on CKM status from nonadvanced to advanced stages. The results of Qgcomp (Table S7) indicated that combined exposure to multiple PAH metabolites in urine was significantly associated with advanced CKM status (OR = 1.12, 95%CI = 1.02, 1.23). Among the PAH exposures, 2‐NAP and 2‐FLU contributed most significantly, with their weights being 0.14 and 0.35, respectively (Figure 4).

Furthermore, the results of the BKMR‐P model are similar to those of Qgcomp. For BKMR‐P, we estimated the posterior mean (Table S4) of the advanced CKM status when six PAH metabolites were set at a particular percentile compared to when five PAH metabolites were all set at their 50th percentile. As shown in Figure 5A, we found that compared with when all PAHs were at the median, as the mixture of six PAHs increased from the 20th percentile to the 80th percentile, the estimated risk of CKM increased, indicating that the mixture of PAHs has a positive combined effect on the development of CKM. Meanwhile, in Figure 5B, we can observe that when other PAHs are fixed at the 25th, 50th, and 75th percentiles, 2‐NAP and 2‐FLU have a positive effect on CKM.

Figure 5BKMR‐P: (A) Joint effect (95% credible interval) of the mixtures on growth indicators. When all polycyclic aromatic hydrocarbons are at a specific percentile, the relationship between polycyclic aromatic hydrocarbon mixtures and the advanced CKM status compared with the values when all polycyclic aromatic hydrocarbons are at their 50th percentile. (B) Single pollutant association (estimates and 95% credible intervals, gray dashed line at the null). This figure compares the late‐stage CKM status when a single pollutant is at the 75th percentile and the 25th percentile and when all other exposures are fixed at the 25th, 50th, or 75th percentile.

**Figure:**
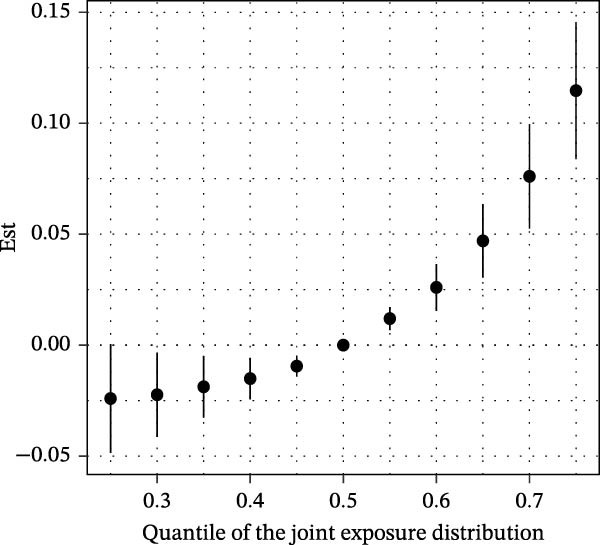
(A)

**Figure:**
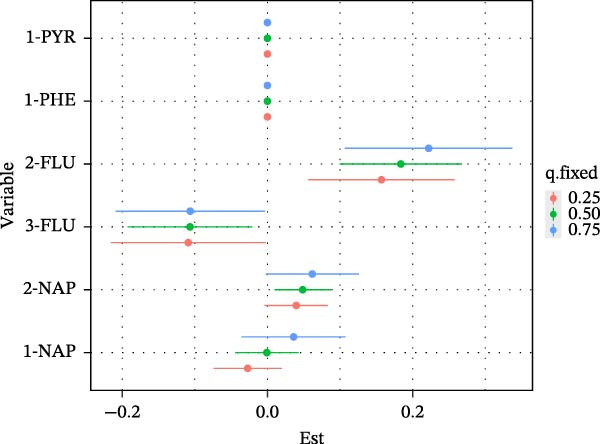
(B)

### 3.4. The Association Between PAHs and RAR

The relationship between all six PAHs and RAR is shown in Table S8. We developed three models. In the third fully adjusted model, all six PAH metabolites were positively correlated with RAR. As for the E–R reaction, all PAH metabolites were meaningful, but only 1‐PHE and 1‐PYR had a linear relationship (Figure S5).

### 3.5. The Association Between RAR and Advanced CKM Status

Logistic regression indicated an extremely strong positive correlation between the progression of RAR and the advanced CKM status. The risk of advanced CKM increases by 22% with each additional SD in RAR.

### 3.6. Mediation Analysis of RAR on Associations of PAHs With Advanced CKM Status

**Figure 6:**
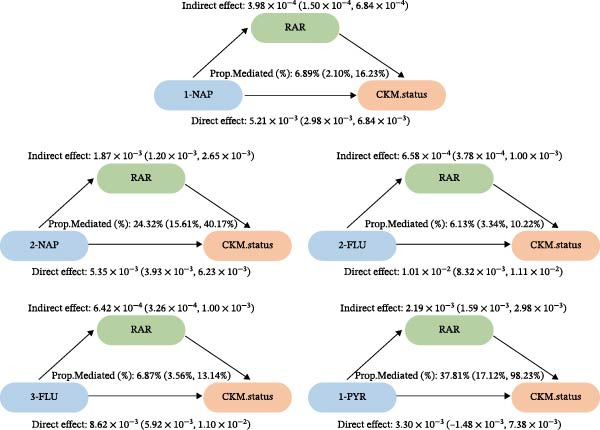
The mediating effect of RAR in the risk relationship between the PAHs and the advanced CKM status.

The results of the mediation analysis indicated that RAR played a mediating role in the development of all five types of PAHs and CKM (Figure 6 and Table S10). The mediation proportions and associated *p*‐Values for each exposure mediator are as follows: 1‐NAP mediated 6.89% (*p* = 0.004) of the association, while 2‐NAP showed a mediation proportion of 24.32% (*p* < 0.001). For 2‐FLU and 3‐FLU, the mediation proportions were 6.13% (*p* < 0.001) and 6.87% (*p* < 0.001), respectively. In contrast, 1‐PHE exhibited no significant mediation, with a proportion of 0.6% (*p* = 0.964). Notably, 1‐PYR suggested the highest mediation proportion at 37.81% (*p* = 0.040).

### 3.7. Associations of Urinary 2‐FLU, 1‐NAP With Advanced CKM Status Stratified by Participant Characteristics

**Figure 7:**
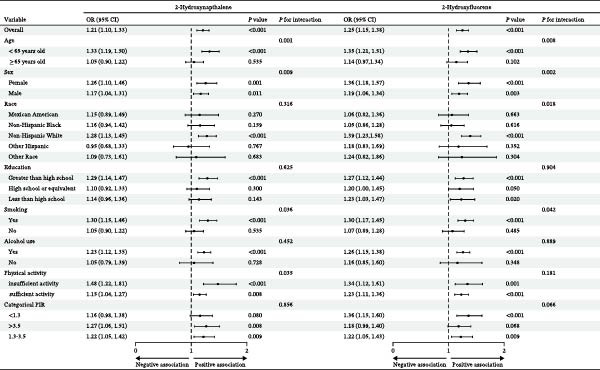
Subgroup analysis of 1‐NAP and 2‐FLU and the advanced CKM status.

As shown in Figure 7, the relationships between 2‐NAP, 2‐FLU, and the advanced CKM status were largely similar in different subgroups. We found that age, sex, and smoking status modulate the association between PAH metabolites in urine and CKM development (*p* interaction <0.05), and the detrimental effects of these two metabolites were more pronounced in younger smokers.

### 3.8. Sensitivity Analysis

To verify the robustness of our conclusion, we adjusted the investigation period and RAR, respectively, on the basis of model 3. Additionally, after imputing missing covariates and excluding left‐censored observations (<LOD), we recalibrated the model to verify the consistency of our findings. When we used noncreatinine‐corrected PAHs as exposure and creatinine as a covariate in the model, the results remained robust. All these correlations remained consistent. For the observed nonlinear relationship between PAHs and the advanced CKM status as well as RAR, the corresponding PAHs were divided into quartiles. The results showed that 2‐FLU, 3‐FLU, and 1‐PYR were positively correlated with the advanced CKM status, and the positive correlations between 1‐NAP, 2‐NAP, 2‐FLU, 3‐FLU, and RAR remained robust (Tables 11–17).

## 4. Discussion

Our study presents novel evidence that urinary PAH metabolites are independently and jointly associated with advanced CKM syndrome status in the general U.S. population. In the logistic regression analysis, 1‐NAP, 2‐NAP, 2‐FLU, and 3‐FLU were associated with a high risk of the advanced CKM status. A significant E–R relationship was established between PAHs and RAR levels as well as the risk of advanced CKM. Most nonlinear E–R relationships indicated that there was a safety threshold for the adverse effects of PAHs on the CKM status. According to the Qgcomp, 2‐NAP and 2‐FLU are the main contributors. Meanwhile, the combined association results through BKMR‐P showed the mixture of urinary PAHs was associated with advanced CKM status. Furthermore, we found that RAR mediates the relationship between various PAHs and the CKM status to the advanced stage. To our knowledge, this is the first study to investigate the independent and combined relationship between PAHs and the CKM status. Crucially, we identified the RAR as a significant mediator in this pathway, explaining 6.13%–37.81% of PAH toxicity. Our research adds epidemiological evidence from the perspectives of public health, environmental pollution control, and clinical patient management.

PAHs are legacy pollutants that have drawn significant public health attention. Many PAHs are highly toxic to humans and possess carcinogenic and mutagenic properties [37]. Several studies using the NHANES dataset have also shown that exposure to PAHs is associated with a variety of chronic diseases. A study from 2021, which utilized data from NHANES from 2005 to 2012, found that PAHs were associated with nonalcoholic fatty liver disease, and lipid metabolism disorders mediated this process [38]. Similarly, another study indicates that PAHs are closely related to childhood obesity [39]. Additionally, this relationship is not only confined to a specific group of people. Previous studies have indicated that this kind of harm also exists in China. Tian et al. [40] found a consistent association between each of the seven individual PAHs and cardiovascular outcomes. Our results extend these observations by establishing PAHs as risk factors for systemic advanced CKM status, with RAR serving as a novel mechanistic bridge. RAR has emerged as a novel and accessible biomarker that reflects systemic inflammation and nutritional status, and its clinical relevance has been studied across various diseases [41]. Recent meta‐analyses and cohort studies consistently demonstrate that higher RAR is associated with increased risk in CVDs, such as acute myocardial infarction, heart failure, stroke, and aortic valvular disease, with a clear exposure‐response relationship [42].

CKM, as a more widespread disease affecting the entire body, conducting research in this population can better reflect the comprehensive impact of PAHs on multiple systems throughout the body. Hyperglycemia, insulin resistance, and lipotoxicity are central, leading to oxidative and endoplasmic reticulum stress, mitochondrial dysfunction, and impaired energy production. RAR may biologically connect PAH exposure to CKM progression because it simultaneously captures two PAH‐responsive processes: (1) systemic oxidative‐inflammatory stress influencing erythrocyte heterogeneity (higher RDW) and (2) inflammation‐related hepatic synthetic dysfunction and malnutrition (lower albumin). Inflammatory responses are mediated by multiple pathways. For instance, PAHs, as agonists of AhR, inhibit SIRT1 by activating AhR [43]. It can weaken the interaction between SIRT1 and NF‐κB and SOD to promote inflammatory responses and oxidative stress [44]. Excess reactive oxygen species can damage erythrocyte membranes and alter erythropoiesis, thereby increasing RDW, and may contribute to microvascular dysfunction and tissue hypoxia, which are relevant to both cardiovascular remodeling and renal injury in CKM [45, 46]. In parallel, liver function impairment caused by PAHs can lead to a decrease in serum albumin [47]. Hypoalbuminemia reflects the malnutrition‐inflammation state and reduces plasma oncotic pressure, promoting vascular leakage and aggravating interstitial edema between the heart and kidneys, potentially accelerating cardiorenal deterioration. By integrating these two signals, RAR may better represent the “inflammation–oxidative stress–nutrition” axis than either component alone, providing a plausible mechanistic bridge linking PAHs to advanced CKM. This is consistent with our mediation models where high‐activity AhR ligands like 1‐PYR exhibited 37.8% mediation via RAR. Moreover, PAHs act as endocrine disruptors, interfering with hormone signaling (notably estrogen and aryl hydrocarbon receptors), which can influence islet cell development and function [48].

Notably, 1‐PHE showed negligible mediation (0.6%, *p* = 0.964), likely due to its unique metabolism via CYP1A2 to inert metabolites, unlike ROS‐generating CYP1B1 substrates (e.g., 1‐PYR) [39, 49]. Furthermore, another advantage of our research is that we have conducted a more detailed exploration of the exposure‐response relationship between PAHs and diseases. We have reviewed previous literature [38, 39, 50, 51], and although this linear or nonlinear relationship has also been found in other diseases, few studies have explored whether there is a safe threshold for the harm of PAHs. Our research found that the four PAH metabolites, 2‐FLU, 3‐FLU, 1‐PHE, and 1‐PYR, have a threshold effect. That is, before this threshold point, their harm to the human body does not seem significant: for 2‐FLU (threshold: 6.35 ng/g Cr), concentrations below this level showed minimal risk (*p* = 0.485), while exceeding it increased CKM risk (OR = 1.66, *p* < 0.001). Similar thresholds existed for 3‐FLU (4.17 ng/g Cr) and 1‐PYR (5.09 ng/g Cr). This finding provides potential reference values for occupational exposure limits.

BKMR‐P mixture analysis quantified real‐world risk: The risk ratio of CKM in individuals at the 80th percentile of combined exposure to PAHs was much higher than that in individuals at the 50th percentile (Figure 5A). 2‐FLU and 2‐NAP were dominant drivers (weights: 0.34 and 0.14), suggesting these metabolites should be prioritized in biomonitoring. It indicates that we should pay close attention to people with high exposure to PAHs, such as workers in industries like coking plants [52] or residents in areas with high urban pollution levels [53, 54]. Notably, the concentration of 2‐NAP has consistently been the highest among various PAH metabolites in multiyear investigations, which may be attributed to increased environmental emissions and sources from common indoor sources such as air fresheners, toilet deodorants, paints, dyes, floors, and carpets, as well as some insecticides [55].

Occupational exposure may influence the observed associations between PAHs and CKM progression. Workers in industries such as coke production, aluminum smelting, petrochemical processing, and asphalt paving can experience substantially higher and more complex PAH exposures than the general population, through both inhalation and dermal routes [56, 57]. In such settings, PAHs have been linked to cardiovascular‐related alterations, including autonomic dysfunction and cardiometabolic risk, suggesting that occupational exposure could act as an effect modifier and potentially amplify associations at higher dose ranges [58]. Conversely, incomplete characterization of occupation and co‐exposures (e.g., particulate matter, metals, shift work, and smoking patterns) may introduce residual confounding, which could bias estimates in either direction.

Subgroup analyses identified specific demographic and lifestyle‐related vulnerabilities to PAH‐induced CKM progression. The stronger associations observed in females may stem from higher lipophilic PAH accumulation and endocrine‐disrupting effects on estrogen signaling [59]. Environmental PAH exposure tends to be higher in younger adults (<65 years), and age has been shown to modify PAH‐associated health risks [60, 61], implying that younger cohorts may be more sensitive to PAH‐related CKM progression. Although a pronounced positive association emerged in non‐Hispanic Whites, this must be interpreted cautiously, as this group comprises ~68% of our cohort; the lack of significance in other races likely reflects insufficient statistical power rather than intrinsic biological differences. Furthermore, unhealthy lifestyles, particularly cigaret smoking and insufficient physical activity, may amplify the adverse effects of PAH exposure through synergistic increases in oxidative stress, inflammation, and reduced cardiometabolic resilience [62], as smoking promotes oxidative and inflammatory damage and physical activity mitigates such processes [63].

Our study possesses several notable strengths. First, to our knowledge, this is the first large‐scale epidemiological investigation to delineate the independent and joint associations of urinary PAHs with advanced CKM syndrome, moving beyond single‐disease focus to capture multisystem pathophysiology. Second, we identified the red cell distribution width‐to‐albumin ratio (RAR) as a novel and clinically accessible mediator, explaining up to 37.8% of the association, which offers a feasible biomarker for early risk stratification. Third, utilizing advanced statistical approaches (RCS, BKMR‐P, and Qgcomp), we not only confirmed exposure‐response relationships but also identified actionable safety thresholds for key PAHs—a finding with direct implications for environmental regulation and occupational health guidelines. Furthermore, our mixture analysis reflects real‐world exposure scenarios, highlighting 2‐NAP and 2‐FLU as priority contaminants. These consistent results across multiple sensitivity analyses and subgroup assessments reinforce the robustness of our conclusions.

There are still several limitations that need to be addressed. Firstly, the cross‐sectional design limits our ability to establish a temporal relationship between PAH exposure, RAR alteration, and CKM progression. CKM status was based on baseline measurements, precluding the observation of intra‐individual longitudinal changes from early to advanced stages. We cannot rule out the possibility of reverse causation—specifically, that advanced CKM status (such as prevalent CKD or CVD) may exacerbate systemic inflammation and nutritional depletion, thereby directly causing an elevation in RAR. Therefore, our mediation analysis should be interpreted as an exploratory evaluation of potential pathways, warranting future longitudinal studies for causal confirmation. Secondly, considering the simultaneous measurement of multiple NHANES periods, we only selected six comparable PAH metabolites. Other PAH metabolites await further study. Thirdly, the half‐life of PAHs in the body is very short, usually only a few hours to tens of hours. Therefore, a single urine test mainly reflects the exposure situation within a few days before sampling. It may not accurately represent long‐term, chronic, or intermittent exposure patterns. When evaluating the association with chronic diseases (such as CKM syndrome), this exposure measurement error may dilute the true effect association. Repeated measurements will be needed in future research to calculate the average cumulative exposure, which will help to assess long‐term health risks more accurately. Fourth, detailed occupational information was unavailable, precluding the identification of participants with potentially high occupational PAH exposure and the execution of related subgroup analyses. Fifth, PAH sources are highly diverse. Exposure levels are heavily influenced by specific occupational settings and residential environmental factors, such as local traffic density. Because these contextual variables can independently affect cardiometabolic and kidney outcomes, the possibility of residual confounding cannot be fully excluded. To build upon our findings, future research should integrate detailed work‐history data, apply job‐exposure matrices, and incorporate geospatial indicators (e.g., residential proximity to traffic) to better characterize high‐exposure subpopulations and isolate the specific effects of distinct PAH sources on CKM progression.

## 5. Conclusion

This study demonstrates that PAHs are significant independent and synergistic contributors to advanced CKM syndrome. Among the PAHs, 2‐NAP and 2‐FLU were identified as primary drivers, with exposure‐response relationships and threshold effects observed. The RAR was established as a novel mediator. These findings highlight the critical role of environmental PAH exposure in metabolic, renal, and cardiovascular health deterioration, emphasizing the clinical utility of RAR as a biomarker and the need for targeted public health interventions to mitigate PAH‐related health risks.

**Nomenclature**
AHA: American Heart Association
AIC: Akaike information criteria
ALT: Alanine aminotransferase
AST: Aspartate aminotransferase
BIC: Bayesian information criteria
BKMR‐P: Bayesian kernel machine regression‐prospective extension
BMI: Body mass index
CI: Confidence interval
CKD: Chronic kidney disease
CKM: Cardiovascular‐kidney‐metabolic syndrome
CVD: Cardiovascular disease
eGFR: Estimated glomerular filtration rate
GLM: Generalized linear model
MET: Metabolic equivalent
MS: Metabolic syndrome
NCEP‐ATP III: National Cholesterol Education Program Adult Treatment Panel III
NHANES: National Health and Nutrition Examination Survey
OR: Odds ratio
PAHs: Polycyclic aromatic hydrocarbon
PIR: Poverty income ratio
Qgcomp: Quantile‐based g‐computation
RAR: Red blood cell distribution width‐to‐albumin ratio
RCS: Restricted cubic spline
ROS: Reactive oxygen species
UACR: Urinary albumin‐to‐creatinine ratio.

## Author Contributions

**Yifeng Huang**: conceptualization, methodology, data analysis, writing – original draft. **Youxia Zhao**: methodology, data curation, writing – original draft. **Qizhang Man**: formal analysis, writing – original draft, visualization. **Song Liu**: data curation, statistical analysis, writing – review and editing. **Ying Yang**: investigation, visualization, writing – review and editing. **Jinfeng Wen**: validation, data curation, writing – review and editing. **Lei Fan**: supervision, project administration, writing – review and editing. **Hao Xie**: conceptualization, supervision, writing – review and editing, correspondence. **Keyun Zhang**: methodology, writing – review and editing, correspondence. **Jing Wang**: supervision, methodology, writing – review and editing, correspondence.

## Funding

This work was supported by the National Natural Science Foundation of China Youth Fund (Grant 82202662, 2022), the China Postdoctoral Science Foundation (Grant 2024T170380, 2024), the Natural Science Foundation of Guangdong Province of China (Grant 2024A1515012767, 2024), and the Guangzhou Science and Technology Planning Project (Grant 2023A04J2314, 2023).

## Ethics Statement

Studies involving human participants, human materials or human data are partially conducted under the Helsinki Announcement. The protocols of NHANES were approved by the institutional review board of the National Center for Health Statistics, CDC (https://www.cdc.gov/nchs/nhanes/about/erb.html). NHANES has obtained written informed consent from all participants.

## Consent

The authors have nothing to report.

## Conflicts of Interest

The authors declare no conflicts of interest.

## Supporting Information

Additional supporting information can be found online in the Supporting Information section.

## Supporting information


**Supporting Information** The supporting information include additional tables and figures that support the main findings of the study. These materials provide detailed data on the definitions of CKM syndrome stages, statistical models used for analysis, and associations between PAH metabolites and CKM status, as well as the mediating role of RAR. Key additional content includes descriptive statistics of PAH metabolites across different NHANES cycles, exposure‐response relationships, and sensitivity analyses. These resources offer a comprehensive view of the data and statistical methods underpinning the study’s conclusions.

## Data Availability

The datasets generated and analyzed in the current study are available at NHANES website: https://wwwn.cdc.gov/nchs/nhanes/Default.aspx.
