# Supplementary material for: Urinary Polycyclic Aromatic Hydrocarbons and Advanced Cardiovascular‐Kidney‐Metabolic Syndrome: Inflammatory‐Nutritional Pathways as Mediators
Source: Mediators Inflamm. 2026 Jun 2;2026:5585916. doi: 10.1155/mi/5585916 (PMC13239313; doi:10.1155/mi/5585916)

**This file includes:**

**Supplement table 1.** The definitions of various disease states of CKM.

**Supplement table 2.** PREVENT equations for 10-year cardiovascular disease (CVD) risk.

**Supplement table 3.** Definition of cardiovascular-kidney-metabolic syndrome staging.

**Supplement table 4.** Summary results from different models.

**Supplement table 5.** Descriptive statistics of six PAHs metabolites in different investigation years.

**Supplement table 6.** Knots selection of restricted cubic spline analysis.

**Supplement table 7.** Quantile g-computation models for the Joint effects of PAHs.

**Supplement table 8.** Associations between urinary PAHs metabolites and RAR.

**Supplement table 9.** Associations between RAR and CKM status.

**Supplement table 10.** Analysis of the mediating effect of RAR-mediated Association between PAHs and CKM.

**Supplement table 11.** Associations of polycyclic aromatic hydrocarbon (PAHs) metabolites with CKM status (adjusting for the NHANES cycle).

**Supplement table 12.** Associations of polycyclic aromatic hydrocarbon (PAHs) metabolites with CKM status (After multiple interpolations).

**Supplement table 13.** Associations of polycyclic aromatic hydrocarbon (PAHs) metabolites with CKM status (adjusting for the RAR).

**Supplement table 14**. Associations of polycyclic aromatic hydrocarbon (PAHs) metabolites with CKM status ((Exclude data that is below the LOD level).

**Supplement table 15.** Associations of polycyclic aromatic hydrocarbon (PAHs) metabolites with CKM status (PAH without correction for creatinine; creatinine as a covariate).

**Supplement table 16.** The association between the quartiles of variables and the CKM status.

**Supplement table 17.** The association between the quartiles of variables and RAR.

**Supplement table 18.** Occurrence and detection limits of polycyclic aromatic hydrocarbons (PAHs).

**Supplement figure 1.** A directed acyclic graph of the association between PAHs metabolites and CKM and the risk of death.

**Supplement figure 2.** The variance inflation factor of exposure and covariates in the model.

**Supplement figure 3.** Sample density curves of PAHs metabolites in the non-advanced group and the advanced group.

**Supplement figure 4.** Correlation analysis among six PAHs metabolites.

**Supplement figure 5.** Dose-response relationship between polycyclic aromatic hydrocarbons and RAR.

**Supplement table 1.** The definitions of various disease states of CKM

| **CKM disease** | **Specific classification** | **Diagnostic criteria** |
| --- | --- | --- |
| CVD | Clinical CVD | Ever had any of the following diseases: stroke, myocardial infarction, angina pectoris, chronic heart failure, coronary heart disease |
|  | Subclinical CVD | Any of the following criterion is met:  1) Very high-risk CKD in KDIGO classification: UACR ≥ 300 mg/g and eGFR ≤ 45-59 ml/min/1.73m2, UACR ≥ 30 mg/g and eGFR ≤ 30-44 ml/min/1.73m2 , or eGFR ≤ 29 ml/min/1.73m.  2) Predicted 10-year CVD risk ≥ 20% (PREVENT equations) |
| Kidney diseases | CKD | Moderate-to-high-risk CKD in KDIGO classification: UACR ≥ 30 mg/g and eGFR ≥ 60 ml/min/1.73m^2^, UACR < 300 mg/g and eGFR ≤ 45-59 ml/min/1.73m^2^, or UACR < 30 mg/g and eGFR ≤ 30-44 ml/min/1.73m^2^. |
| Metabolic disorders | Overweight/obesity | BMI ≥25 kg/m^2^ (or ≥23 kg/m2 if Asian ancestry) * |
|  | Abdominal obesity | Waist circumference ≥88/102 cm in female/male (or if Asian ancestry ≥80/90 cm in female/male) |
|  | Prediabetes | Fasting blood glucose ≥ 100-124 mg/dL or HbA1c ≥ 5.7%-6.4% and without self-reported diagnosis of diabetes, use of insulin, or oral hypoglycemic agents |
|  | Diabetes | Fasting blood glucose ≥ 125 mg/dL or HbA1c ≥ 6.5% or self-reported diagnosis of diabetes, use of insulin, or oral hypoglycemic drugs |
|  | Hypertension | SBP ≥130 mmHg or DBP ≥80 mmHg or self-reported diagnosis of hypertension or use of antihypertensive medications |
|  | Hypertriglyceridemia | Triglycerides ≥ 135 mg/dL |
|  | MetS | MetS is defined by the presence of 3 or more of the following:  1) Waist circumference ≥88/102 cm in female/male (or if Asian ancestry ≥80/90 cm in female/male).  2) HDL cholesterol ＜50/40 mg/dL in female/male.  3) Triglycerides ≥150 mg/dL.  4) Elevated blood pressure (SBP ≥130 mmHg or DBP ≥80 mmHg and/or use of antihypertensive medications)  5) Fasting blood glucose ≥100 mg/dL |
| BMI, body mass index; HbA1c, glycated hemoglobin; SBP, systolic blood pressure; DBP, diastolic blood pressure; HDL-C, high-density lipoprotein cholesterol; MetS, metabolic syndrome; CKD, chronic kidney disease; KDIGO, The Kidney Disease: Improving Global Outcomes; UACR, urinary albumin to creatinine ratio; eGFR, estimated glomerular filtration rate; CVD, cardiovascular disease. | | |

**Supplement table 2.** PREVENT equations for 10-year cardiovascular disease (CVD) risk

| Male | **log-Odds** = -3.031168 + 0.7688528 × (age – 55) /10 + 0.0736174 × (TC – HDL-C – 3.5) – 0.0954431 × (HDL-C – 1.3) /0.3 – 0.4347345 × (min(SBP, 110) – 110) /20 + 0.3362658 × (max(SBP, 110) – 130) /20 + 0.7692857 × (if diabetes) + 0.4386871 × (if current smoker) + 0.5378979 × (min(eGFR, 60) – 60) / -15 + 0.0164827 × (max(eGFR, 60) – 90) / -15 + 0.288879 × (if using anti-hypertensive medication) – 0.1337349 × (if using statin) – 0.0475924 × (if using anti-hypertensive medication) × (max(SBP, 110) – 130) /20 + 0.150273 × (if using statin) × (TC – HDL-C – 3.5) – 0.0517874 × (age – 55) /10 × (TC – HDL-C – 3.5) + 0.0191169 × (age – 55) /10 × (HDL-C – 1.3) /0.3 – 0.1049477 × (age – 55) /10 × (max(SBP, 110) – 130) /20 – 0.2251948 × (age – 55) /10 × (if diabetes) – 0.0895067 × (age – 55) /10 × (if current smoker) – 0.1543702 × (age – 55) /10 × (min(eGFR, 60) – 60) / -15  **Risk** = exp(log-Odds) / (1 + exp(log-Odds)) |
| --- | --- |
| Female | **log-Odds** = -3.307728 + 0.7939329 × (age – 55) /10 + 0.0305239 × (TC – HDL-C – 3.5) – 0.1606857 × (HDL-C – 1.3) /0.3 – 0.2394003 × (min(SBP, 110) – 110) /20 + 0.360078 × (max(SBP, 110) – 130) /20 + 0.8667604 × (if diabetes) + 0.5360739 × (if current smoker) + 0.6045917 × (min(eGFR, 60) – 60) / -15 + 0.0433769 × (max(eGFR, 60) – 90) / -15 + 0.3151672 × (if using anti-hypertensive medication) – 0.1477655 × (if using statin) – 0.0663612 × (if using anti-hypertensive medication) × (max(SBP, 110) – 130) /20 + 0.1197879 × (if using statin) × (TC – HDL-C – 3.5) – 0.0819715 × (age – 55) /10 × (TC – HDL-C – 3.5) + 0.0306769 × (age – 55) /10 × (HDL-C – 1.3) /0.3 – 0.0946348 × (age – 55) /10 × (max(SBP, 110) – 130) /20 – 0.27057 × (age – 55) /10 × (if diabetes) – 0.078715 × (age – 55) /10 × (if current smoker) – 0.1637806 × (age – 55) /10 × (min(eGFR, 60) – 60) / -15  **Risk** = exp(log-Odds) / (1 + exp(log-Odds)) |
| Abbreviations: TC, total cholesterol; HDL-C, high-density lipoprotein cholesterol; SBP, systolic blood pressure; eGFR, estimated glomerular filtration rate. | |

**Supplement table 3.** Definition of cardiovascular-kidney-metabolic syndrome staging.

| CKM syndrome stages | Definition |
| --- | --- |
| CKM syndrome stage 0 | Individuals with normal BMI and waist circumference, normoglycemia, normotension, a normal lipid profile, and no evidence of CKD or subclinical or clinical CVD. |
| CKM syndrome stage 1 | Individuals with overweight/obesity, abdominal obesity, or adipose tissue dysfunction without hypertension, prediabetes, diabetes, MetS, CKD, or subclinical/clinical CVD. |
| CKM syndrome stage 2 | Individuals with any of hypertriglyceridemia, hypertension, diabetes, MetS, moderate-to-high-risk CKD. |
| CKM syndrome stage 3 | Presence of very-high-risk KDIGO CKD stages or a high-estimated 10-year CVD risk. The 10-year CVD risk was assessed using the AHA PREVENT equations for predicting cardiovascular events. A 10-year CVD risk of 20% or greater was classified as high risk. |
| CKM syndrome stage 4 | Clinical CVD (self-reported diagnosed cardiovascular disease, including coronary heart disease, heart failure, angina, heart attack, or stroke). |

Abbreviations: AHA, American Heart Association; CKM, cardiovascular-kidney-metabolic; BMI, body mass index; CKD, chronic kidney disease; CVD, cardiovascular disease; MetS, metabolic syndrome; KDIGO, The Kidney Disease: Improving Global Outcomes;

**Supplement table 4.**Summary results from different models

|  | CKM Progression | | | |
| --- | --- | --- | --- | --- |
| PAHs | Logistic Regression | RCS | BKMR-P (PIP) | Qgcomp  (weight) |
| **1-Hydroxynapthalene** | + | + | 1 | 0.02 |
| **2-Hydroxynapthalene** | **+** | **+** | 1 | 0.14 |
| **1-Hydroxypyrene** | - | + | 0 | -0.01 |
| **2-Hydroxyfluorene** | + | + | 0.12 | 0.34 |
| **3-Hydroxyfluorene** | **+** | **+** | 0.88 | -0.21 |
| **1-Hydroxyphenanthrene** | - | + | 0 | -0.1 |

Note: Restricted cubic splines (RCS); Quantile-based g-Computation (Qgcomp); Probit extension of Bayesian Kernel Machine Regression (BKMR-P); posterior inclusion probability (PIP); “+” means positive association between PAHs and outcomes; “-” means negative association between PAHs and outcomes.

**Supplement table 5.** Descriptive statistics of six PAHs metabolites in different investigation years

| **Cycle** | **1-Hydroxynapthalene**  **mean (25%-75%)** | **2-Hydroxynapthalene**  **mean (25%-75%)** | **1-Hydroxypyrene**  **mean (25%-75%)** | **2-Hydroxyfluorene**  **mean (25%-75%)** | **3-Hydroxyfluorene**  **mean (25%-75%)** | **1-Hydroxyphenanthrene**  **mean (25%-75%)** |
| --- | --- | --- | --- | --- | --- | --- |
| **2001-2002** | 7.66 (6.74 - 8.49) | 7.88 (7.05 - 8.68) | 3.83 (3.23 - 4.40) | 5.77 (5.04 - 6.45) | 4.9 (4.07 - 5.71) | 4.89 (4.38 - 5.36) |
| **2003-2004** | 7.93 (6.86 - 8.98) | 8.11 (7.28 - 8.96) | 4.38 (3.69 - 5.04) | 5.74 (4.92 - 6.58) | 4.87 (3.86 - 5.93) | 4.97 (4.48 - 5.41) |
| **2005-2006** | 7.96 (6.71 - 9.07) | 8.29 (7.5 - 9.05) | 4.51 (3.82 - 5.15) | 5.8 (4.96 - 6.56) | 4.87 (3.88 - 5.77) | 4.98 (4.46 - 5.41) |
| **2007-2008** | 7.93 (6.85 - 8.93) | 8.39 (7.67 - 9.09) | 4.72 (4.12 - 5.25) | 5.78 (4.99 - 6.49) | 4.82 (3.89 - 5.69) | 4.93 (4.42 - 5.4) |
| **2009-2010** | 7.85 (6.8 - 8.85) | 8.36 (7.63 - 9.06) | 4.86 (4.26 - 5.42) | 5.66 (4.86 - 6.33) | 4.74 (3.84 - 5.55) | 4.93 (4.45 - 5.38) |
| **2011-2012** | 7.64 (6.51 - 8.57) | 8.54 (7.83 - 9.2) | 4.8 (4.26 - 5.29) | 5.68 (4.94 - 6.22) | 4.73 (3.89 - 5.41) | 4.93 (4.43 - 5.38) |
| **2013-2014** | 7.62 (6.62 - 8.52) | 8.61 (7.95 - 9.28) | 5.02 (4.5 - 5.52) | 5.45 (4.72 - 6.04) | 4.64 (3.77 - 5.39) | 4.69 (4.23 - 5.14) |
| **2015-2016** | 7.56 (6.51 - 8.5) | 8.72 (8.07 - 9.37) | 4.91 (4.34 - 5.39) | 5.45 (4.71 - 6.01) | 4.63 (3.75 - 5.36) | 4.73 (4.25 - 5.14) |

**Supplement table 6.** Knots selection of restricted cubic spline analysis

| **Variable** | **Knots** | **AIC** | **BIC** | **Variable** | **Knots** | **AIC** | **BIC** |
| --- | --- | --- | --- | --- | --- | --- | --- |
| **2-Hydroxyfluorene** | 3 | 3,291.55 | 3,438.47 | **1-Hydroxynapthalene** | 3 | 3,310.95 | 3,457.87 |
| **2-Hydroxyfluorene** | 4 | 3,293.36 | 3,447.62 | **1-Hydroxynapthalene** | 4 | 3,313.07 | 3,467.33 |
| **2-Hydroxyfluorene** | 5 | 3,294.52 | 3,456.14 | **1-Hydroxynapthalene** | 5 | 3,308.12 | 3,469.73 |
| **2-Hydroxyfluorene** | 6 | 3,296.70 | 3,465.66 | **1-Hydroxynapthalene** | 6 | 3,291.10 | 3,460.06 |
| **3-Hydroxyfluorene** | 3 | 3,293.69 | 3,440.61 | **2-Hydroxynapthalene** | 3 | 3,303.94 | 3,450.86 |
| **3-Hydroxyfluorene** | 4 | 3,295.50 | 3,449.77 | **2-Hydroxynapthalene** | 4 | 3,306.03 | 3,460.30 |
| **3-Hydroxyfluorene** | 5 | 3,297.31 | 3,458.92 | **2-Hydroxynapthalene** | 5 | 3,305.39 | 3,467.00 |
| **3-Hydroxyfluorene** | 6 | 3,298.80 | 3,467.76 | **2-Hydroxynapthalene** | 6 | 3,303.77 | 3,472.72 |
| **1-Hydroxyphenanthrene** | 3 | 3,316.91 | 3,463.83 | **1-Hydroxypyrene** | 3 | 3,308.03 | 3,454.95 |
| **1-Hydroxyphenanthrene** | 4 | 3,318.44 | 3,472.70 | **1-Hydroxypyrene** | 4 | 3,309.07 | 3,463.34 |
| **1-Hydroxyphenanthrene** | 5 | 3,317.83 | 3,479.44 | **1-Hydroxypyrene** | 5 | 3,311.06 | 3,472.67 |
| **1-Hydroxyphenanthrene** | 6 | 3,317.32 | 3,486.28 | **1-Hydroxypyrene** | 6 | 3,312.72 | 3,481.67 |

**Supplement table 7.** Quantile g-computation models for the Joint effects of PAHs.

|  | **OR (95% CI)** | ***P*-value** |
| --- | --- | --- |
| **CKM status** | 1.12 (1.02, 1.23) | **0.016** |

Model was adjusted for age, sex, race, education, marital status, poverty income ratio (PIR), body mass index (BMI), smoking, alcohol use, aspartate aminotransferase (AST), glutamate aminotransferase (ALT), and physical activity level (MET).

**Supplement table 8.** Associations between urinary PAHs metabolites and standardized RAR

|  | **Model 1** | | | **Model 2** | | | **Model 3** | | |
| --- | --- | --- | --- | --- | --- | --- | --- | --- | --- |
| **Characteristic** | **Beta** | **95% CI**^1^ | ***P*-value** | **Beta** | **95% CI**^1^ | ***P*-value** | **Beta** | **95% CI**^1^ | ***P*-value** |
| **1-Hydroxynapthalene** | 0.03 | 0.02, 0.05 | **<0.001** | 0.01 | 0.00, 0.03 | **0.028** | 0.03 | 0.01, 0.04 | **<0.001** |
| **2-Hydroxynapthalene** | 0.13 | 0.12, 0.15 | **<0.001** | 0.10 | 0.08, 0.12 | **<0.001** | 0.10 | 0.08, 0.13 | **<0.001** |
| **1-Hydroxypyrene** | 0.07 | 0.05, 0.09 | **<0.001** | 0.08 | 0.06, 0.10 | **<0.001** | 0.13 | 0.10, 0.15 | **<0.001** |
| **2-Hydroxyfluorene** | 0.04 | 0.02, 0.06 | **<0.001** | 0.04 | 0.02, 0.05 | **<0.001** | 0.07 | 0.04, 0.09 | **<0.001** |
| **3-Hydroxyfluorene** | 0.02 | 0.01, 0.04 | **0.004** | 0.03 | 0.01, 0.04 | **<0.001** | 0.07 | 0.05, 0.09 | **<0.001** |
| **1-Hydroxyphenanthrene** | 0.02 | -0.01, 0.04 | 0.240 | -0.01 | -0.03, 0.02 | 0.657 | 0.03 | 0.00, 0.06 | **0.042** |
| ^1^CI = Confidence Interval | | | | | | | | | |
| Model 1 did not adjust for confounding factors, and the Model 2 adjusted for demographic factors. The Model 3 further adjusted for body mass index (BMI), smoking and alcohol use, aspartate aminotransferase (AST), glutamate aminotransferase (ALT), and physical activity level (MET). | | | | | | | | | |

**Supplement table 9.** Associations between standardized RAR and CKM status

| **Characteristic** | **Model** | **OR (95% CI)** | ***P*-value** |
| --- | --- | --- | --- |
| **RAR** | Model 1 | 1.50 (1.43, 1.58) | **<0.001** |
|  | Model 2 | 1.40 (1.32, 1.50) | **<0.001** |
|  | Model 3 | 1.22 (1.11, 1.35) | **<0.001** |
| ^1^OR = Odds Ratio, CI = Confidence Interval  RAR, Red blood cell distribution width albumin ratio  Model 1 did not adjust for confounding factors, and the Model 2 adjusted for demographic factors. The Model 3 further adjusted for body mass index (BMI), smoking and alcohol use, aspartate aminotransferase (AST), glutamate aminotransferase (ALT), and physical activity level (MET). | | | |

**Supplement table 10.** Analysis of the mediating effect of RAR-mediated Association between PAHs and CKM

| **Variables** | **Total effect** | **Direct effect** | **Indirect effect** | **Mediation proportion (%)** |
| --- | --- | --- | --- | --- |
| **1-Hydroxynapthalene** | 5.62×10^-3^ (3.27×10^-3^, 7.23×10^-3^), **P<0.001** | 5.21×10^-3^ (2.98×10^-3^, 6.84×10^-3^), **P<0.001** | 3.98×10^-4^ (1.50×10^-4^, 6.84×10^-4^), **P=0.002** | 6.89 (2.10, 16.23), **P=0.004** |
| **2-Hydroxynapthalene** | 7.37×10^-3^ (6.44×10^-3^, 7.86×10^-3^), **P<0.001** | 5.35×10^-3^ (3.93×10^-3^, 6.23×10^-3^), **P<0.001** | 1.87×10^-3^ (1.20×10^-3^, 2.65×10^-3^), **P<0.001** | 24.32 (15.61, 40.17), **P<0.001** |
| **1-Hydroxypyrene** | 5.53×10^-3^ (9.87×10^-4^, 9.33×10^-3^), P=0.018 | 3.30×10^-3^ (-1.48×10^-3^, 7.38×10^-3^), P=0.178 | 2.19×10^-3^ (1.59×10^-3^, 2.98×10^-3^), **P<0.001** | 37.81 (17.12, 98.23), **P=0.040** |
| **2-Hydroxyfluorene** | 1.08×10^-2^ (9.23×10^-3^, 1.18×10^-2^), **P<0.001** | 1.01×10^-2^ (8.32×10^-3^, 1.11×10^-2^), **P<0.001** | 6.58×10^-4^ (3.78×10^-4^, 1.00×10^-3^), **P<0.001** | 6.13 (3.34, 10.222), **P<0.001** |
| **3-Hydroxyfluorene** | 9.30×10^-3^ (6.62×10^-3^, 1.16×10^-2^), **P<0.001** | 8.62×10^-3^ (5.92×10^-3^, 1.10×10^-2^), **P<0.001** | 6.42×10^-4^ (3.26×10^-4^, 1.00×10^-3^), **P<0.001** | 6.87 (3.56, 13.14), **P<0.001** |
| **1-Hydroxyphenanthrene** | 3.72×10^-4^ (-7.59×10^-3^, 6.77×10^-3^), P=0.870 | 4.91×10^-4^ (-7.37×10^-3^, 6.82×10^-3^), P=0.846 | -1.19×10^-4^ (-8.24×10^-4^, 5.35×10^-4^), P=0.738 | 0.64 (-95.13, 98.31), P=0.964 |
| Note: β(95% CI), p value  Adjusted for demographic-related variables | | | | |

**Supplement table 11.** Associations of polycyclic aromatic hydrocarbon (PAHs) metabolites with CKM status (adjusting for the NHANES cycle)

| **Characteristic** | **Model** | | |
| --- | --- | --- | --- |
|  | **OR**^1^ | **95% CI**^1^ | ***P*-value** |
| **1-Hydroxynapthalene** | 1.09 | 1.03, 1.14 | **0.001** |
| **2-Hydroxynapthalene** | 1.15 | 1.07, 1.23 | **<0.001** |
| **1-Hydroxypyrene** | 1.08 | 1.00, 1.17 | 0.056 |
| **2-Hydroxyfluorene** | 1.19 | 1.11, 1.27 | **<0.001** |
| **3-Hydroxyfluorene** | 1.15 | 1.08, 1.22 | **<0.001** |
| **1-Hydroxyphenanthrene** | 1.03 | 0.95, 1.12 | 0.504 |
| ^1^OR = Odds Ratio, CI = Confidence Interval  Model was adjusted for age, sex, race, education, marital status, poverty income ratio (PIR), body mass index (BMI), smoking, alcohol use, aspartate aminotransferase (AST), glutamate aminotransferase (ALT), and physical activity level (MET). | | | |

**Supplement table 12**. Associations of polycyclic aromatic hydrocarbon (PAHs) metabolites with CKM status (After multiple interpolations)

|  | **Model** | | |
| --- | --- | --- | --- |
| **Characteristic** | **OR**^1^ | **95% CI**^1^ | ***P*-value** |
| **1-Hydroxynapthalene** | 1.09 | 1.04, 1.14 | **<0.001** |
| **2-Hydroxynapthalene** | 1.12 | 1.05, 1.20 | **<0.001** |
| **1-Hydroxypyrene** | 1.06 | 0.99, 1.13 | 0.112 |
| **2-Hydroxyfluorene** | 1.16 | 1.08, 1.23 | **<0.001** |
| **3-Hydroxyfluorene** | 1.09 | 1.03, 1.15 | **0.003** |
| **1-Hydroxyphenanthrene** | 1.03 | 0.95, 1.12 | 0.445 |
| ^1^OR = Odds Ratio, CI = Confidence Interval  Model was adjusted for age, sex, race, education, marital status, poverty income ratio (PIR), body mass index (BMI), smoking, alcohol use, aspartate aminotransferase (AST), glutamate aminotransferase (ALT), and physical activity level (MET). | | | |

**Supplement table 13**. Associations of polycyclic aromatic hydrocarbon (PAHs) metabolites with CKM status ((adjusting for the RAR)

|  | **Model** | | |
| --- | --- | --- | --- |
| **Characteristic** | **OR**^1^ | **95% CI**^1^ | ***P*-value** |
| **1-Hydroxynapthalene** | 1.10 | 1.03, 1.17 | **0.003** |
| **2-Hydroxynapthalene** | 1.19 | 1.09, 1.31 | **<0.001** |
| **1-Hydroxypyrene** | 1.04 | 0.94, 1.15 | 0.430 |
| **2-Hydroxyfluorene** | 1.24 | 1.13, 1.36 | **<0.001** |
| **3-Hydroxyfluorene** | 1.17 | 1.08, 1.27 | **<0.001** |
| **1-Hydroxyphenanthrene** | 1.08 | 0.96, 1.22 | 0.199 |
| ^1^OR = Odds Ratio, CI = Confidence Interval  Model was adjusted for age, sex, race, education, marital status, poverty income ratio (PIR), body mass index (BMI), smoking, alcohol use, aspartate aminotransferase (AST), glutamate aminotransferase (ALT), and physical activity level (MET), RAR. | | | |

**Supplement table 14**. Associations of polycyclic aromatic hydrocarbon (PAHs) metabolites with CKM status ((Exclude data that is below the LOD level)

|  | **Model** | | |
| --- | --- | --- | --- |
| **Characteristic** | **OR**^1^ | **95% CI**^1^ | ***P*-value** |
| **1-Hydroxynapthalene** | 1.10 | 1.04, 1.17 | **0.002** |
| **2-Hydroxynapthalene** | 1.22 | 1.11, 1.35 | **<0.001** |
| **1-Hydroxypyrene** | 1.09 | 0.98, 1.20 | 0.111 |
| **2-Hydroxyfluorene** | 1.25 | 1.14, 1.37 | **<0.001** |
| **3-Hydroxyfluorene** | 1.18 | 1.09, 1.28 | **<0.001** |
| **1-Hydroxyphenanthrene** | 1.08 | 0.96, 1.22 | 0.216 |
| ^1^OR = Odds Ratio, CI = Confidence Interval  Model was adjusted for age, sex, race, education, marital status, poverty income ratio (PIR), body mass index (BMI), smoking, alcohol use, aspartate aminotransferase (AST), glutamate aminotransferase (ALT), and physical activity level (MET), RAR. | | | |

**Supplement table 15**. Associations of polycyclic aromatic hydrocarbon (PAHs) metabolites with CKM status (PAH without correction for creatinine; creatinine as a covariate)

|  | **Model** | | |
| --- | --- | --- | --- |
| **Characteristic** | **OR**^1^ | **95% CI**^1^ | ***P*-value** |
| **1-Hydroxynapthalene** | 1.08 | 1.03, 1.12 | **<0.001** |
| **2-Hydroxynapthalene** | 1.14 | 1.07, 1.22 | **<0.001** |
| **1-Hydroxypyrene** | 1.06 | 0.99, 1.13 | 0.117 |
| **2-Hydroxyfluorene** | 1.17 | 1.10, 1.25 | **<0.001** |
| **3-Hydroxyfluorene** | 1.13 | 1.07, 1.20 | **<0.001** |
| **1-Hydroxyphenanthrene** | 1.07 | 0.99, 1.16 | 0.098 |
| ^1^OR = Odds Ratio, CI = Confidence Interval  Model was adjusted for age, sex, race, education, marital status, poverty income ratio (PIR), body mass index (BMI), smoking, alcohol use, aspartate aminotransferase (AST), glutamate aminotransferase (ALT), and physical activity level (MET), creatinine. | | | |

**Supplement table 16.** The association between the quartiles of variables and the CKM status

|  | **Model 1** | | | **Model 2** | | | **Model 3** | | |
| --- | --- | --- | --- | --- | --- | --- | --- | --- | --- |
| **Characteristic** | **OR**^1^ | **95% CI**^1^ | ***P*-value** | **OR**^1^ | **95% CI**^1^ | ***P*-value** | **OR**^1^ | **95% CI**^1^ | ***P*-value** |
| **2-Hydroxyfluorene** |  |  |  |  |  |  |  |  |  |
| Q1 (0.78-4.88) | — | — |  | — | — |  | — | — |  |
| Q2 (4.88-5.39) | 0.86 | 0.74, 1.00 | 0.051 | 1.00 | 0.84, 1.20 | 0.959 | 0.97 | 0.77, 1.24 | 0.836 |
| Q3 (5.39-6.32) | 0.97 | 0.83, 1.12 | 0.656 | 1.11 | 0.94, 1.33 | 0.225 | 1.16 | 0.91, 1.47 | 0.223 |
| Q4 (6.32-10.32) | 1.14 | 0.98, 1.31 | 0.080 | 1.53 | 1.28, 1.82 | **<0.001** | 1.89 | 1.48, 2.42 | **<0.001** |
| **3-Hydroxyfluorene** |  |  |  |  |  |  |  |  |  |
| Q1 (-0.34-3.86) | — | — |  | — | — |  | — | — |  |
| Q2 (3.86-4.42) | 0.69 | 0.60, 0.81 | **<0.001** | 0.80 | 0.67, 0.95 | **0.012** | 0.90 | 0.71, 1.14 | 0.365 |
| Q3 (4.42-5.58) | 0.76 | 0.66, 0.88 | **<0.001** | 0.88 | 0.74, 1.05 | 0.156 | 1.00 | 0.79, 1.27 | 0.997 |
| Q4 (5.58-9.87) | 0.89 | 0.77, 1.02 | 0.102 | 1.28 | 1.08, 1.53 | **0.005** | 1.76 | 1.38, 2.26 | **<0.001** |
| **1-Hydroxypyrene** |  |  |  |  |  |  |  |  |  |
| Q1 (-0.14-4.03) | — | — |  | — | — |  | — | — |  |
| Q2 (4.03-4.63) | 0.73 | 0.63, 0.84 | **<0.001** | 0.91 | 0.77, 1.07 | 0.247 | 0.92 | 0.73, 1.16 | 0.467 |
| Q3 (4.63-5.26) | 0.65 | 0.56, 0.75 | **<0.001** | 0.93 | 0.78, 1.10 | 0.383 | 0.84 | 0.66, 1.08 | 0.179 |
| Q4 (5.26-9.36) | 0.68 | 0.59, 0.79 | **<0.001** | 1.17 | 0.99, 1.39 | 0.072 | 1.32 | 1.03, 1.68 | **0.028** |
| **1-Hydroxyphenanthrene** |  |  |  |  |  |  |  |  |  |
| Q1 (-0.33-4.38) | — | — |  | — | — |  | — | — |  |
| Q2 (4.38-4.83) | 0.80 | 0.69, 0.93 | **0.004** | 0.78 | 0.65, 0.93 | **0.006** | 0.77 | 0.60, 0.98 | **0.037** |
| Q3 (4.83-5.33) | 0.93 | 0.81, 1.08 | 0.357 | 0.92 | 0.77, 1.10 | 0.353 | 0.88 | 0.69, 1.12 | 0.295 |
| Q4 (5.33-9.89) | 1.07 | 0.93, 1.24 | 0.340 | 1.0 | 0.84, 1.18 | 0.952 | 1.12 | 0.88, 1.42 | 0.362 |
| ^1^OR = Odds Ratio, CI = Confidence Interval | | | | | | | | | |
| Model 1 did not adjust for confounding factors, and the Model 2 adjusted for demographic factors. The Model 3 further adjusted for poverty income ratio (PIR), body mass index (BMI), smoking and alcohol use, aspartate aminotransferase (AST), glutamate aminotransferase (ALT), and physical activity level (MET). | | | | | | | | | |

**Supplement table 17.** The association between the quartiles of variables and RAR

|  | **Model 1** | | | **Model 2** | | | **Model 3** | | |
| --- | --- | --- | --- | --- | --- | --- | --- | --- | --- |
| **Characteristic** | **Beta** | **95% CI**^1^ | ***P-*value** | **Beta** | **95% CI**^1^ | ***P*-value** | **Beta** | **95% CI**^1^ | ***P*-value** |
| **1-Hydroxynapthalene** |  |  |  |  |  |  |  |  |  |
| Q1 (3.38-6.69) | — | — |  | — | — |  | — | — |  |
| Q2 (6.69-7.48) | -0.02 | -0.04, 0.01 | 0.189 | -0.04 | -0.06, -0.02 | **<0.001** | 0.00 | -0.03, 0.02 | 0.785 |
| Q3 (7.48-8.74) | -0.02 | -0.04, 0.00 | 0.082 | -0.05 | -0.07, -0.03 | **<0.001** | -0.02 | -0.04, 0.00 | 0.110 |
| Q4 (8.74-17.49) | 0.04 | 0.02, 0.07 | **<0.001** | 0.01 | -0.01, 0.03 | 0.317 | 0.06 | 0.03, 0.08 | **<0.001** |
| **2-Hydroxynapthalene** |  |  |  |  |  |  |  |  |  |
| Q1 (4.53-7.62) | — | — |  | — | — |  | — | — |  |
| Q2 (7.62-8.35) | 0.06 | 0.04, 0.08 | **<0.001** | 0.03 | 0.01, 0.06 | **0.003** | 0.02 | 0.00, 0.05 | 0.094 |
| Q3 (8.35-9.14) | 0.10 | 0.07, 0.12 | **<0.001** | 0.06 | 0.04, 0.09 | **<0.001** | 0.05 | 0.02, 0.07 | **<0.001** |
| Q4 (9.14-12.68) | 0.16 | 0.13, 0.18 | **<0.001** | 0.12 | 0.10, 0.14 | **<0.001** | 0.12 | 0.10, 0.15 | **<0.001** |
| **2-Hydroxyfluorene** |  |  |  |  |  |  |  |  |  |
| Q1 (0.78-4.88) | — | — |  | — | — |  | — | — |  |
| Q2 (4.88-5.39) | -0.01 | -0.03, 0.02 | 0.559 | -0.02 | -0.04, 0.00 | 0.093 | -0.01 | -0.03, 0.02 | 0.607 |
| Q3 (5.39-6.32) | 0.01 | -0.02, 0.03 | 0.610 | -0.01 | -0.03, 0.02 | 0.574 | 0.01 | -0.01, 0.03 | 0.364 |
| Q4 (6.32-10.32) | 0.04 | 0.02, 0.07 | **<0.001** | 0.04 | 0.02, 0.06 | **<0.001** | 0.08 | 0.05, 0.10 | **<0.001** |
| **3-Hydroxyfluorene** |  |  |  |  |  |  |  |  |  |
| Q1 (-0.34-3.86) | — | — |  | — | — |  | — | — |  |
| Q2 (3.86-4.42) | -0.04 | -0.06, -0.02 | **<0.001** | -0.04 | -0.06, -0.01 | **0.001** | -0.01 | -0.03, 0.01 | 0.370 |
| Q3 (4.42-5.58) | -0.03 | -0.06, -0.01 | **0.005** | -0.04 | -0.06, -0.01 | **0.001** | 0.01 | -0.02, 0.03 | 0.676 |
| Q4 (5.58-9.87) | 0.01 | -0.01, 0.03 | 0.344 | 0.02 | 0.00, 0.05 | **0.039** | 0.08 | 0.06, 0.11 | **<0.001** |
| ^1^CI = Confidence Interval. Note: The Beta coefficients and 95% CIs have been multiplied by 10 for better readability. | | | | | | | | | |
| Model 1 did not adjust for confounding factors, and the Model 2 adjusted for demographic factors. The Model 3 further adjusted for poverty income ratio (PIR), body mass index (BMI), smoking and alcohol use, aspartate aminotransferase (AST), glutamate aminotransferase (ALT), and physical activity level (MET). | | | | | | | | | |

**Supplement table 18.** Occurrence and detection limits of polycyclic aromatic hydrocarbons (PAHs)

| **Compound** | **Detection Frequency (%)** | **LOD (ng/L)^1^** |
| --- | --- | --- |
| **1-Hydroxynapthalene** | 99.92 | 44 (2009-2012), 60 (2013-2016) |
| **2-Hydroxynapthalene** | 100.00 | 42 (2009-2012), 90 (2013-2016) |
| **1-Hydroxypyrene** | 90.75 | 10 (2009-2012), 70 (2013-2016) |
| **2-Hydroxyfluorene** | 99.99 | 10 (2009-2012), 8 (2013-2016) |
| **3-Hydroxyfluorene** | 98.82 | 10 (2009-2012), 8 (2013-2016) |
| **1-Hydroxyphenanthrene** | 99.70 | 10 (2009-2012), 9 (2013-2016) |

^1^LOD, limit of detection

Data for 2001–2002 are not included due to the unavailability of official detection results. A consistent approach was applied across all periods to handle values below the limit of detection (LOD).

**Supplement figure 1.** A directed acyclic graph of the association between PAHs metabolites and CKM and the risk of death.
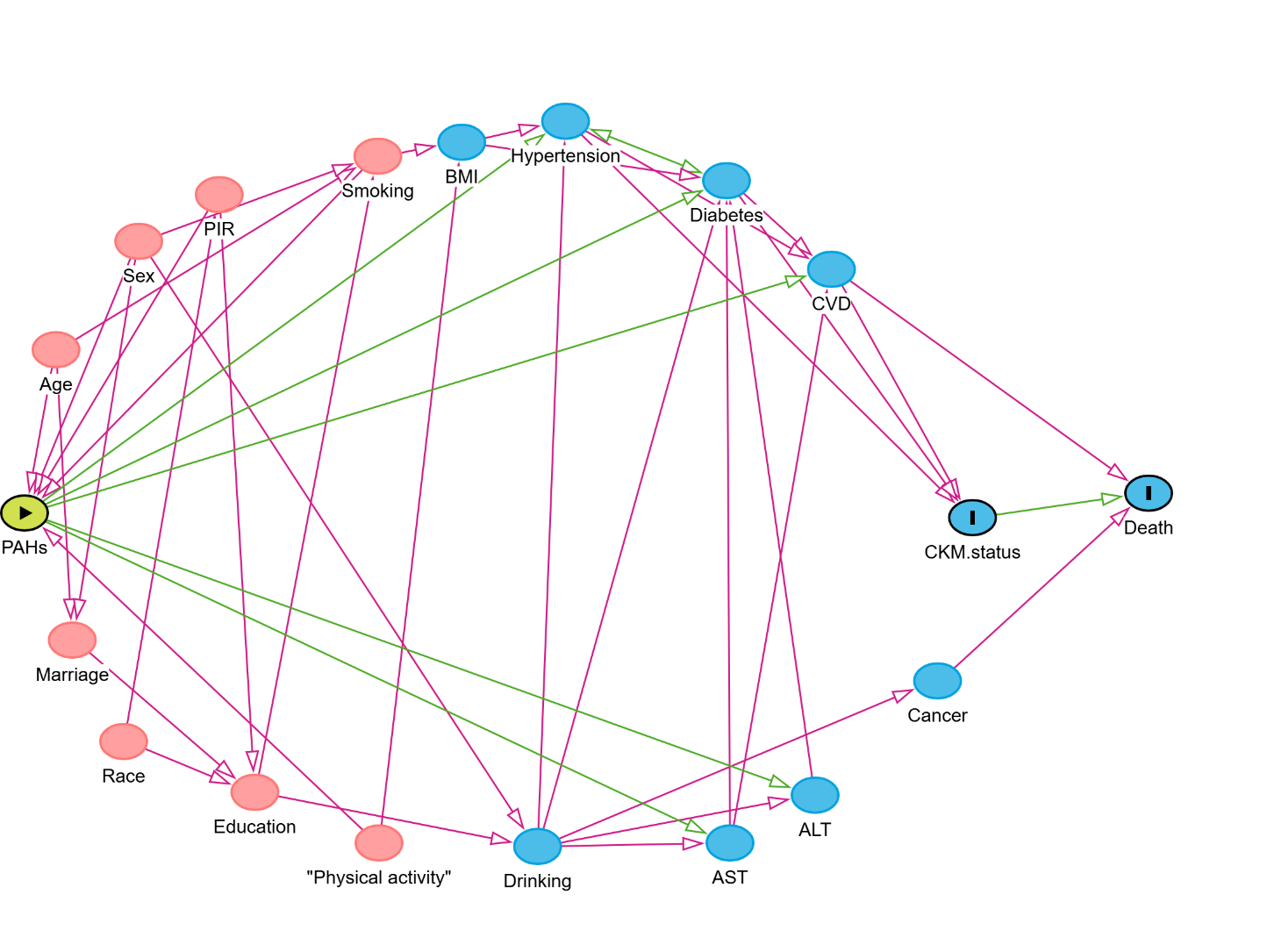


**Supplement figure 2.** The variance inflation factor of exposure and covariates in the model


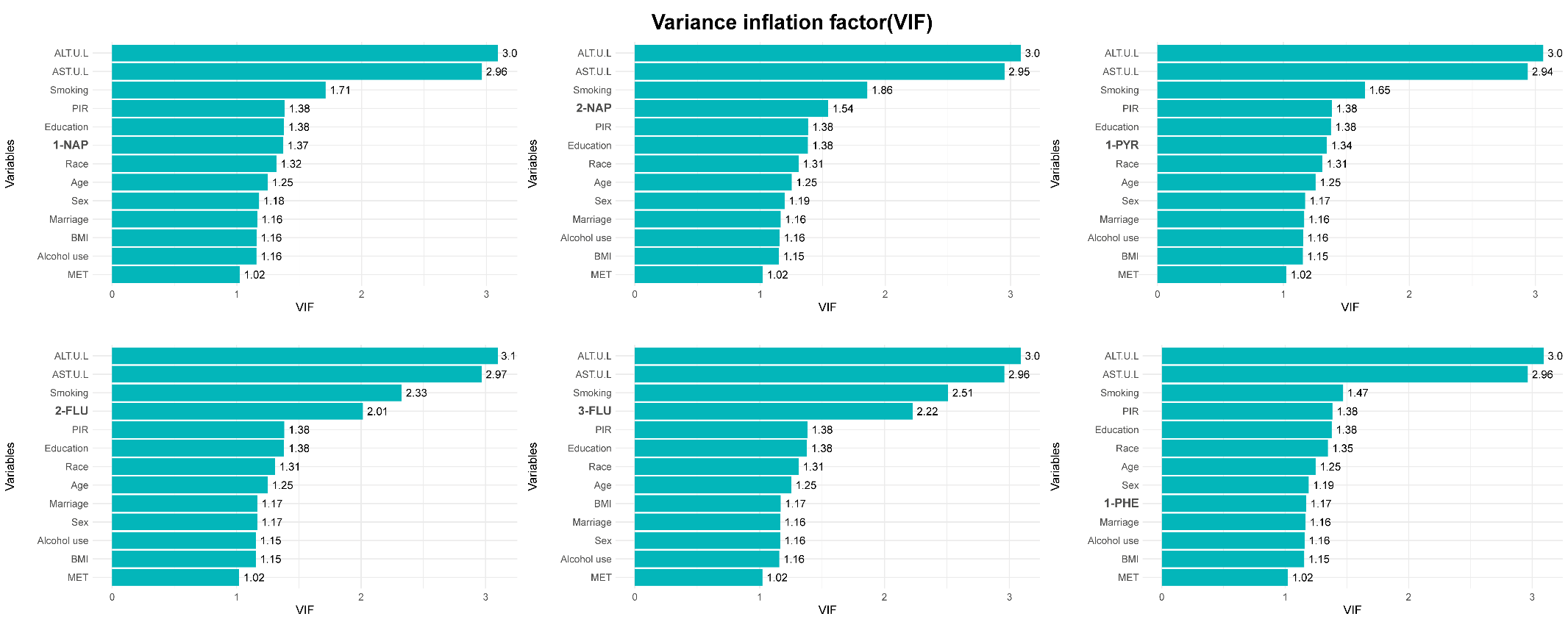


**Supplement figure 3.** Sample density curves of PAHs metabolites in the non-advanced group and the advanced group


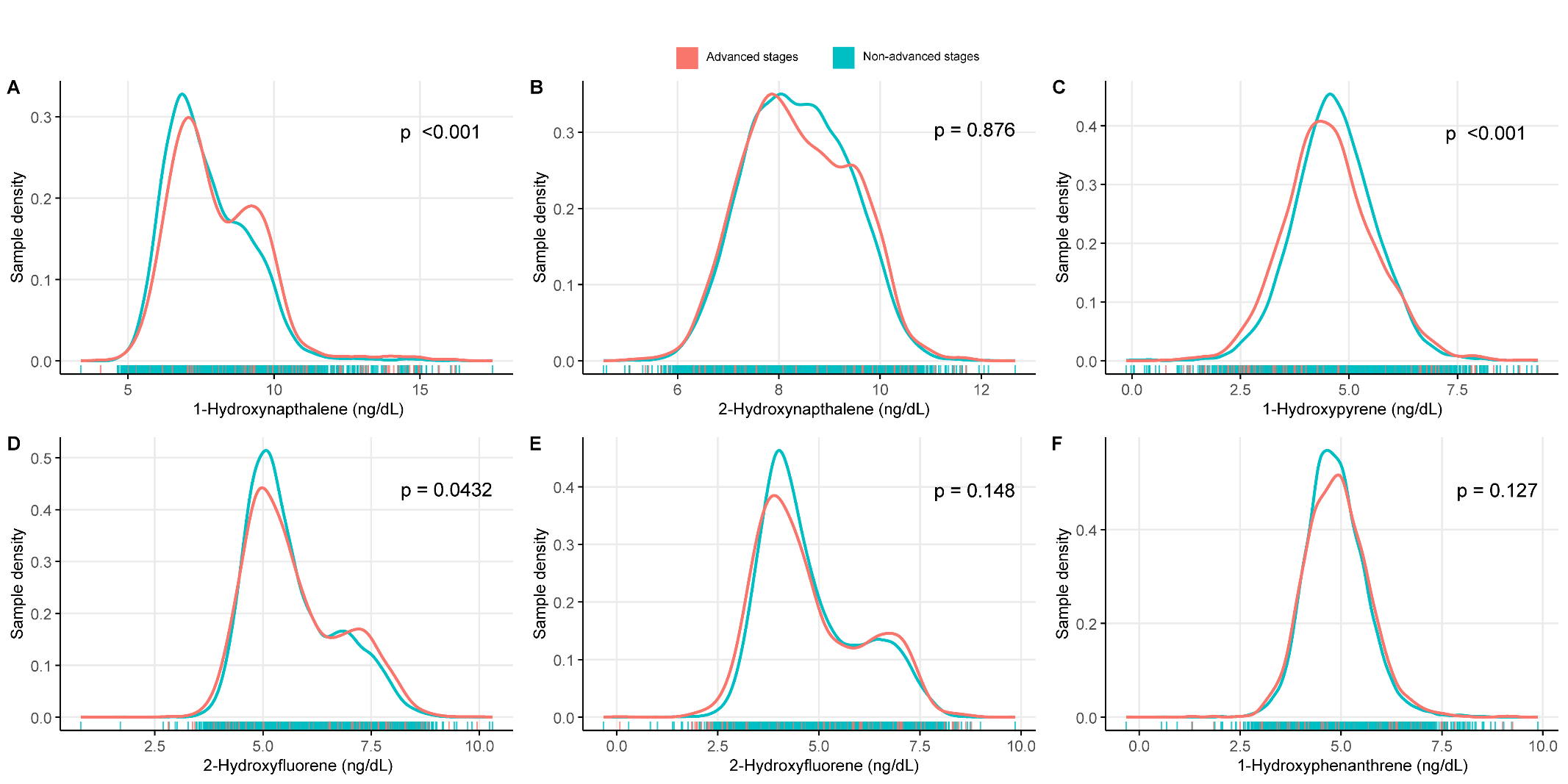


**Supplement figure 4.** Correlation analysis among six PAHs metabolites**
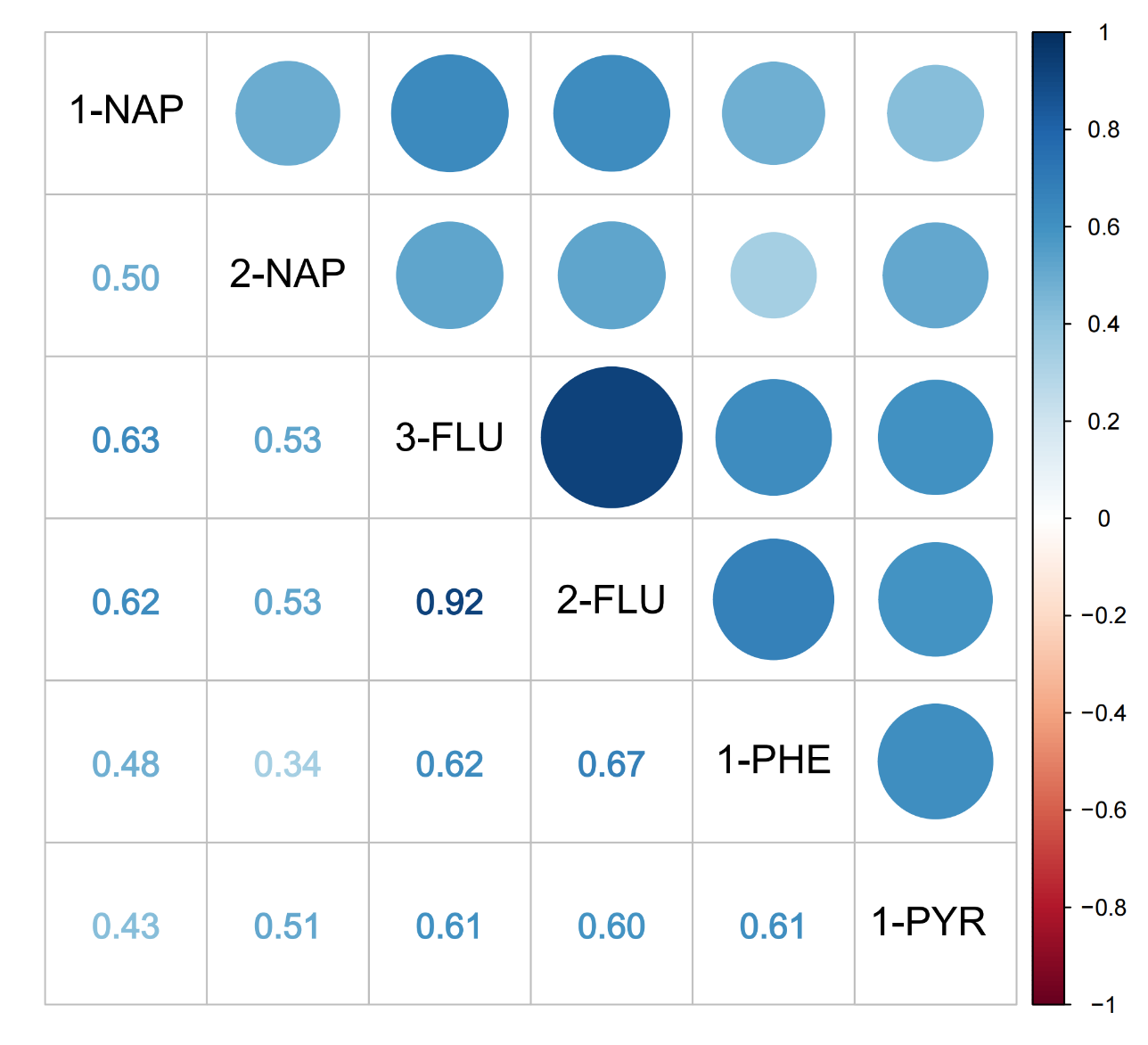
**

**Supplement figure 5.** Dose-response relationship between polycyclic aromatic hydrocarbons and RAR


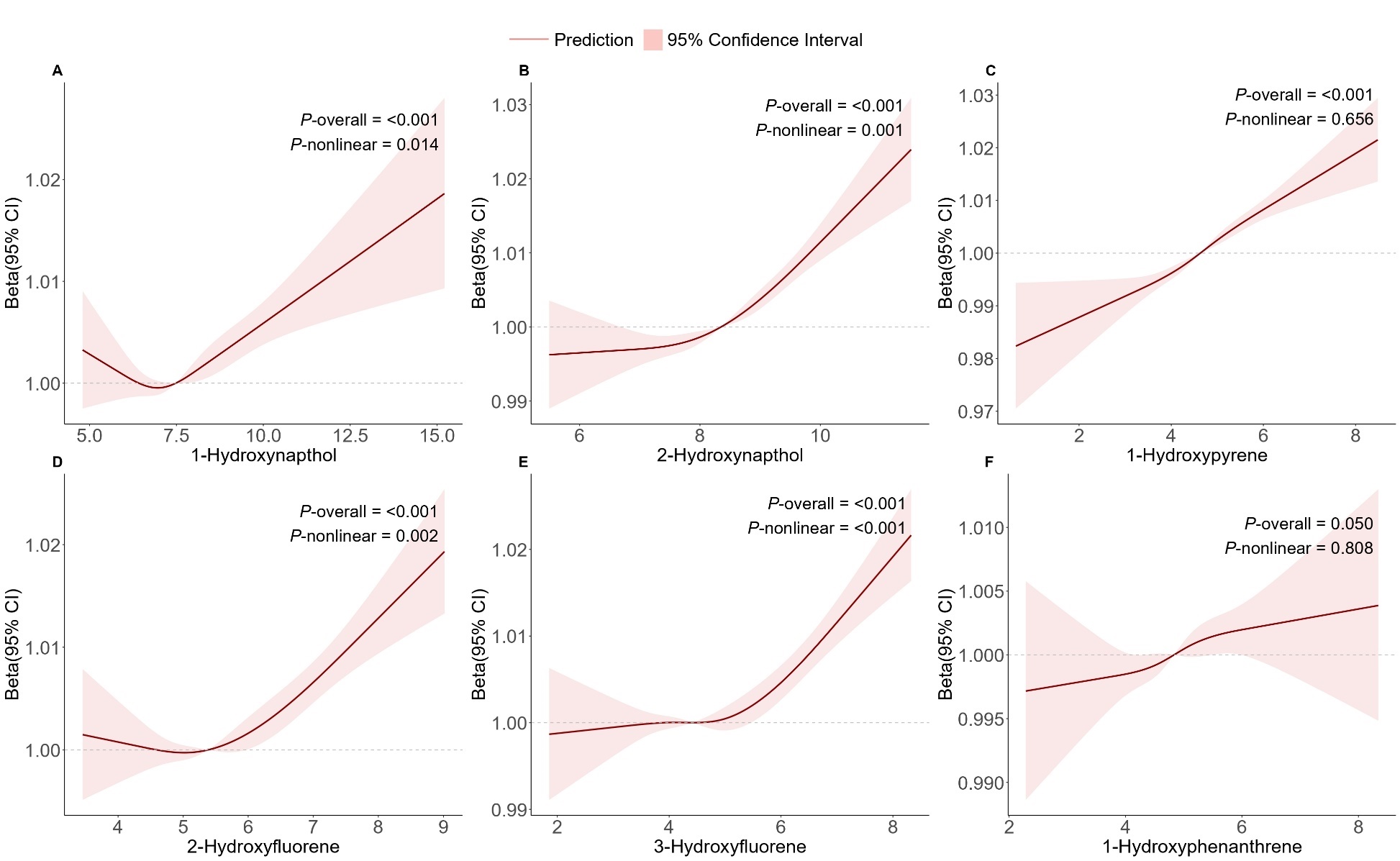

Supplement: Supplementary file 1 — Supporting Information The supporting information include additional tables and figures that support the main findings of the study. These materials provide detailed data on the definitions of CKM syndrome stages, statistical models used for analysis, and associations between PAH metabolites and CKM status, as well as the mediating role of RAR. Key additional content includes descriptive statistics of PAH metabolites across different NHANES cycles, exposure‐response relationships, and sensitivity analyses. These resources offer a comprehensive view of the data and statistical methods underpinning the study’s conclusions. [file MI-2026-5585916-s001.docx]
